# Antigenic Properties of the Human Immunodeficiency Virus Envelope Glycoprotein Gp120 on Virions Bound to Target Cells

**DOI:** 10.1371/journal.ppat.1004772

**Published:** 2015-03-25

**Authors:** Meron Mengistu, Krishanu Ray, George K. Lewis, Anthony L. DeVico

**Affiliations:** 1 The Institute of Human Virology of the University of Maryland School of Medicine, Baltimore, Maryland, United States of America; 2 Center for Fluorescence Spectroscopy of the University of Maryland School of Medicine, Baltimore, Maryland, United States of America; Vanderbilt University School of Medicine, UNITED STATES

## Abstract

The HIV-1 envelope glycoprotein, gp120, undergoes multiple molecular interactions and structural rearrangements during the course of host cell attachment and viral entry, which are being increasingly defined at the atomic level using isolated proteins. In comparison, antigenic markers of these dynamic changes are essentially unknown for single HIV-1 particles bound to target cells. Such markers should indicate how neutralizing and/or non-neutralizing antibodies might interdict infection by either blocking infection or sensitizing host cells for elimination by Fc-mediated effector function. Here we address this deficit by imaging fluorescently labeled CCR5-tropic HIV-1 pseudoviruses using confocal and superresolution microscopy to track the exposure of neutralizing and non-neutralizing epitopes as they appear on single HIV-1 particles bound to target cells. Epitope exposure was followed under conditions permissive or non-permissive for viral entry to delimit changes associated with virion binding from those associated with post-attachment events. We find that a previously unexpected array of gp120 epitopes is exposed rapidly upon target cell binding. This array comprises both neutralizing and non-neutralizing epitopes, the latter being hidden on free virions yet capable of serving as potent targets for Fc-mediated effector function. Under non-permissive conditions for viral entry, both neutralizing and non-neutralizing epitope exposures were relatively static over time for the majority of bound virions. Under entry-permissive conditions, epitope exposure patterns changed over time on subsets of virions that exhibited concurrent variations in virion contents. These studies reveal that bound virions are distinguished by a broad array of both neutralizing and non-neutralizing gp120 epitopes that potentially sensitize a freshly engaged target cell for destruction by Fc-mediated effector function and/or for direct neutralization at a post-binding step. The elucidation of these epitope exposure patterns during viral entry will help clarify antibody-mediated inhibition of HIV-1 as it is measured in vitro and in vivo.

## Author Summary

A major strategy for blocking HIV-1 infection is to target antiviral antibodies or drugs to sites of vulnerability on the surface proteins of the virus. It is a relatively straightforward matter to explore these sites on the surfaces of free HIV-1 particles or on isolated viral envelope antigens. However, one difficulty presented by HIV-1 is that its surface proteins are flexible and change shape once the virus has attached to its host cell. To date, it has been difficult to predict how cell-bound HIV-1 exposes its sites of vulnerability. Yet the antiviral activities of certain antibodies indirectly suggest that there must be unique sites on cell-bound HIV-1 that are not found on free virus. Here, we use new techniques and tools to determine how HIV-1 exposes unique sites of vulnerability after attaching to host cells. We find that the virus exposes a remarkable array of these sites, including ones previously believed hidden. These exposure patterns explain the antiviral activities of various anti-HIV-1 antibodies and provide a new view of how HIV-1 might interact with the immune system. Our study also provides insights for how to target HIV-1 with antiviral antibodies, vaccines, or antiviral agents.

## Introduction

The attachment and entry steps in the Human immunodeficiency virus 1 (HIV-1) replication process involve sequential interactions between viral envelope glycoprotein trimers and cell surface receptors [1]. Each interaction causes conformational alterations in the envelope structure that in turn enables a subsequent phase in the process [2–6]. Attachment begins when the gp120 component of the envelope trimer binds to cell surface CD4. This causes the trimer to assume a structure (CD4-induced or CD4i) that allows gp120 to bind a co-receptor, typically CCR5 in the context of natural virus transmission [7–12]. Co-receptor engagement causes additional conformational rearrangements that translate to the gp41 viral transmembrane glycoprotein, which enables HIV-1-driven membrane fusion and viral entry. HIV-1 envelope-receptor interactions can drive membrane fusion between infected and uninfected cells or virions and target cells. The latter is thought to occur either by direct fusion with target cell membranes; by fusion with membranes of endocytotic vesicles [13, 14]; or by a combination of such processes [15], depending on the microenvironment in which the virus-cell interaction occurs [13].

Numerous experiments with isolated HIV-1 envelope proteins or HIV-driven membrane fusion systems have suggested that the HIV-1 envelope experiences significant changes in epitope presentation as it progresses through the course of HIV-1 attachment and entry [16–21]. These patterns of epitope exposure define the key determinants for HIV-1 susceptibility to the antiviral effects of anti-envelope humoral immunity.

A great deal of effort has been applied toward elucidating conserved neutralizing domains expressed on free virions prior to host cell attachment. In gp120, the most broadly reactive domains include the CD4 binding site [22–29], sequences encompassing a high mannose cluster (2G12 [30]) and glycosylated regions of V1V2 loop structures [31–38]. Other highly conserved epitopes exist within gp120 but are poorly antigenic on free virions. These include the co-receptor binding site and other (CD4i) domains that are fully exposed only after reaction of gp120 with soluble CD4 [39–52].

Very little is known about the antigenic nature of HIV-1 virions residing on the surfaces of permissive cells although several lines of evidence suggest that they are linked with antibody-mediated antiviral activities. A number of studies including our own have shown that CD4+, CCR5+ cells coated with gp120 or whole viral particles are susceptible to Fc-receptor dependent, antibody-mediated antiviral activities, such as antibody-dependent cellular cytotoxicity (ADCC) [53–55] or antibody-dependent cell-mediated viral inhibition (ADCVI) [56], or trogocytosis [57–59]. It is particularly noteworthy that CD4i epitopes enable ADCC against cell-bound virions [55] even though they are hidden within the trimers on free virions as measured by numerous approaches [24, 48, 51, 60–67]. These findings indicate that cell-bound virions exhibit unique and unexpected epitope profiles linked with the development of humoral anti-envelope responses, some of which have antiviral activity. As examples, multiple studies have linked ADCC against gp120 with protection from infection in non-human primate (NHP) models [68–77]; with reduced risk of infection in the RV144 vaccine trial [78]; with decreased risk of mother-to-child HIV-1 transmission [79]; and lower viral loads during HIV disease progression [54, 80, 81]. These observations that CD4i epitopes are involved in humoral effector functions conflict with evidence that CD4i epitopes on viral trimers are occluded from antibodies by steric constraints extant before and after virion attachment to target cells [43, 82–84].

To reconcile this question, we developed microscopy-based methods to interrogate the timing, duration and extent of gp120 epitope exposure as a consequence of virus-cell surface interactions. We focused on virions located on the outer membrane surfaces of freshly targeted cells as they are the most likely candidates for substantive interactions with the anti-HIV-1 humoral response. We find that an array of conserved gp120 domains comprising ADCC targets and CD4i domains are rapidly exposed on cell-bound virions along with constitutively exposed neutralizing domains. Such epitope exposure profiles provide insights for understanding relationships between HIV-1 replication and previously reported humoral immune functions.

## Results

Analyses of HIV-1 virions bound to cells requires a system capable of reflecting changes in epitope exposure and viral protein disposition within the context of certain in vivo conditions and/or in vitro assays commonly used to assess infection. To construct such a system, HIV-1_JRFL_ virions were produced with SNAP-ICAM-1 and CLIP-Vpr fusion proteins (SNAP-ICAM-1/CLIP-Vpr particles) that can be conjugated to Alexa Fluor substrates. After conjugation, the relative dispositions of virion membrane and internal components can be interpreted based on the SNAP-ICAM-1 and CLIP-Vpr fluorescent signals, respectively (see Methods). Further, relationships between these signals should distinguish various populations of bound virions as well as reflect the dynamics of viral replication. For example, CLIP-Vpr signals are expected to decline relative to SNAP-ICAM-1 signals as surface membrane fusion and virus-cell content mixing occurs. Against this background, antibody staining should reveal concurrent patterns of epitope exposure on the HIV-1 envelope.

### Epitope exposure on unbound HIV-1_JRFL_ virions

To define epitope exposure patterns specific to bound virions, it was first necessary to establish which epitopes are exposed on unliganded HIV-1_JRFL_ virions. Previously we used FCS to do this with untagged (i.e., without SNAP-ICAM-1 or CLIP-Vpr) virions in solution [51]. Briefly, this system is based on molecular diffusion rates, which are proportional to the cube root of molecular weight. As a consequence, virion-bound antibody diffuses much more slowly (*D*_*b*_ ~ 8μm^2^/sec) than free antibody (*D*_*nb*_ ~ 65μm^2^/sec). The proportion of a fluorescence-tagged antibody that ‘slows down’ in the presence of a much larger virus species reveals the extent cognate epitope exposure on HIV-1 surface trimers. These studies showed that CD4i epitopes (e.g. 17b) are poorly exposed except when virions are treated with soluble CD4 (sCD4). Other CD4i epitopes such as A32 remain unexposed in the presence and absence of sCD4, in accordance with earlier studies [16, 17].

Similar experiments were carried out to confirm that the SNAP-ICAM-1/CLIP-Vpr-tagged particles expressed the same epitope patterns seen with untagged virions. Alexa Fluor 647-conjugated antibodies were used as probes for this purpose. These included Mab A32 [85–88] against a CD4i epitope in the gp120 C1 domain, Mab C11 [85, 89] against a CD4i epitope in the gp120 C5 domain, Mab 17b [88, 90] against a CD4i epitope in the co-receptor binding site, Mab b12 [23, 91–93] against a constitutively expressed epitope in the CD4 binding site and Mab 2G12 [30, 94–97] against an N-linked glycan (N332) in the outer domain of gp120. An anti-*respiratory syncytial virus* (RSV) antibody, Synagis, was used as a negative control for nonspecific Mab binding.

**Fig 1:**
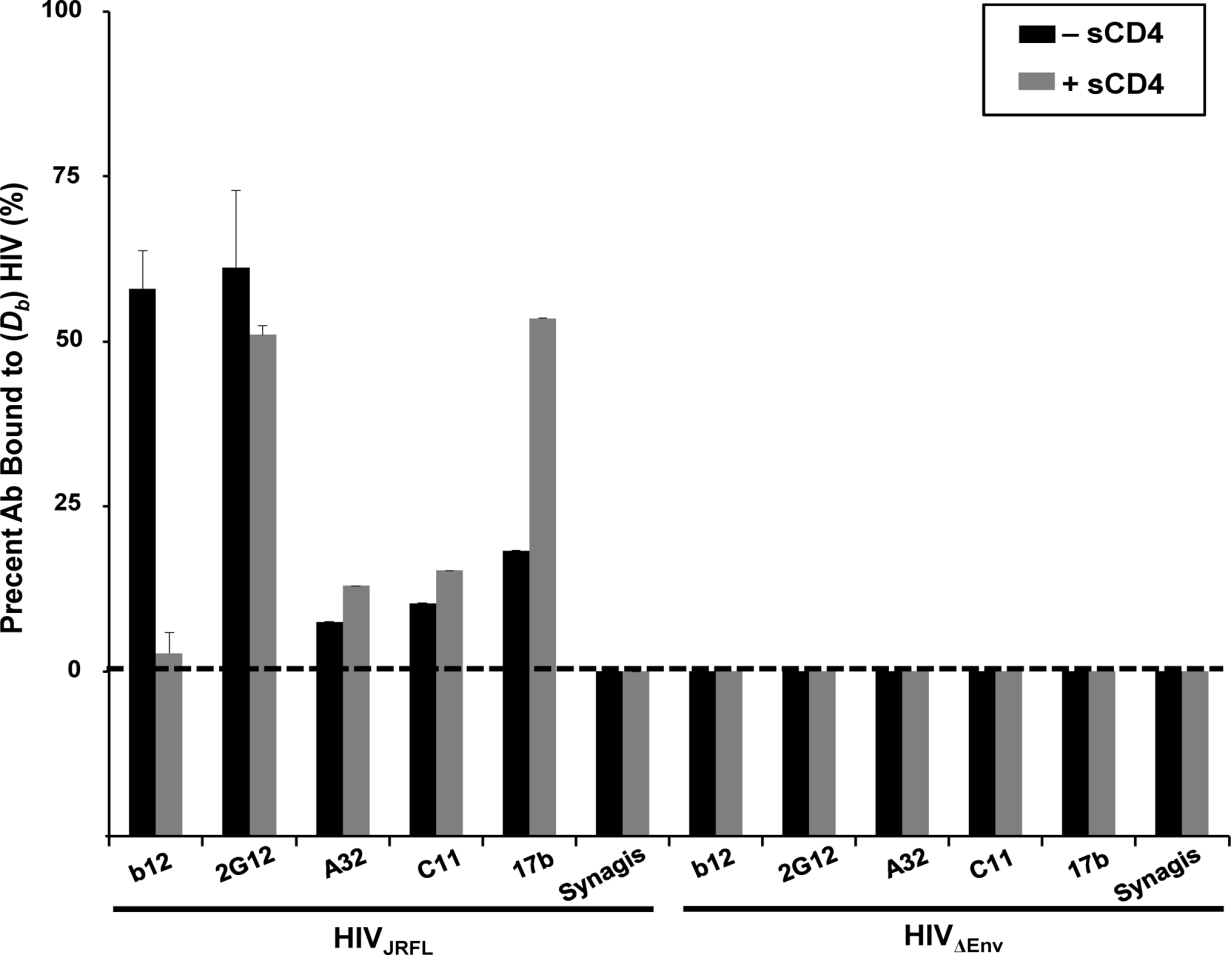
Anti-envelope mAb binding to HIV_JRFL_ virions in solution measured by Fluorescence Correlation Spectroscopy (FCS). HIV_JRFL_ virions (10μg/ml p24 equivalent concentrations) were allowed to interact in solution with Alexa Fluor 647-conjugated test Mabs b12, 2G12, A32, C11, and 17b, or negative control antibody Synagis in a 100μL volume at 4.5–6.6μg/ml final concentrations (see Methods). The relative fraction of Mabs that adopts a slower diffusion coefficient (*D*_*b*_~8 μm^2^ /sec) as a result of virion binding is indicated by black bars. The percentage of Mab binding to virions treated with 100μg/ml sCD4 is shown in grey bars. As a control for nonspecific binding, Mabs were tested for interactions with particles not expressing HIV Env (HIV_ΔENV_). All experiments were repeated at least four times, and average values are shown. Error bars indicate standard deviation.

Only a minor fraction (< 20%) of Mabs C11, A32 and 17b exhibited slower diffusion coefficients indicative of virion binding (Fig. 1), in accordance with our previous findings [51]. Mab 17b binding was markedly improved in the presence of 100μg/ml sCD4 (~50%), while binding signals for Mabs A32 and C11 were not substantially increased, as predicted by previous studies [16]. In comparison, a large fraction (> 50%) of Mabs b12 and 2G12 exhibited virion-bound diffusion coefficients; such binding for Mab b12 was competitively reduced by sCD4. As expected, none of the Mabs exhibited binding to virions devoid of envelope (HIV-1_ΔEnv_); Synagis showed no binding to either type of particle.

Matching observations were made with HIV-1_JRFL_ virions attached to Poly-L-Lysine-coated cover glass probed with Alexa Fluor 488-labeled Mabs (representative images shown in S1 Fig). The virions were fluorescently tagged via SNAP-ICAM-1 and CLIP-Vpr using, respectively, membrane-impermeable Surface Alexa Fluor 546 and membrane-permeable CLIP-Cell Alexa 360 as described in **Methods**. The possibility of interference from free gp120 was addressed by tests with conjugated polyclonal D7324 immunoglobulin, which marks the presence of free gp120. Incubation of the substrate-bound virions with this reagent failed to produce binding signals (representative image in S2A Fig) indicating that free gp120 was unlikely to be a confounding factor. In comparison, Mab binding signals were clearly seen for the b12 and 2G12 epitopes, whereas no staining was seen with Mabs A32 and C11 (S1 Fig). Weak Mab 17b staining was seen on a subset of substrate-captured virions. This variance with FCS is likely to be a peculiar aspect of the capture format, which can be less accurate for probing epitope exposure versus solution binding [51].

### Confocal imaging of single virions bound to target cells

Fluorescently tagged HIV-1_JRFL_ virions (Alexa 546 labeled-SNAP-ICAM-1; Alexa 360-labeled CLIP-Vpr) were incubated with either TZM-bl cells (CD4+; CCR5+) or HeLa-CD4 cells (CD4+; CCR5–) for 0 to 240 minutes at 37°C or 4°C, then washed, fixed with 4% paraformaldehyde, stained with Alexa Fluor 488-conjugated test Mab, and examined by confocal microscopy (see Methods). TZM-bl cells are derived from HeLa cells [98–101] but typically express 10-fold higher surface levels of CD4 versus HeLa-CD4 cells as determined by flow cytometry.

**Fig 2:**
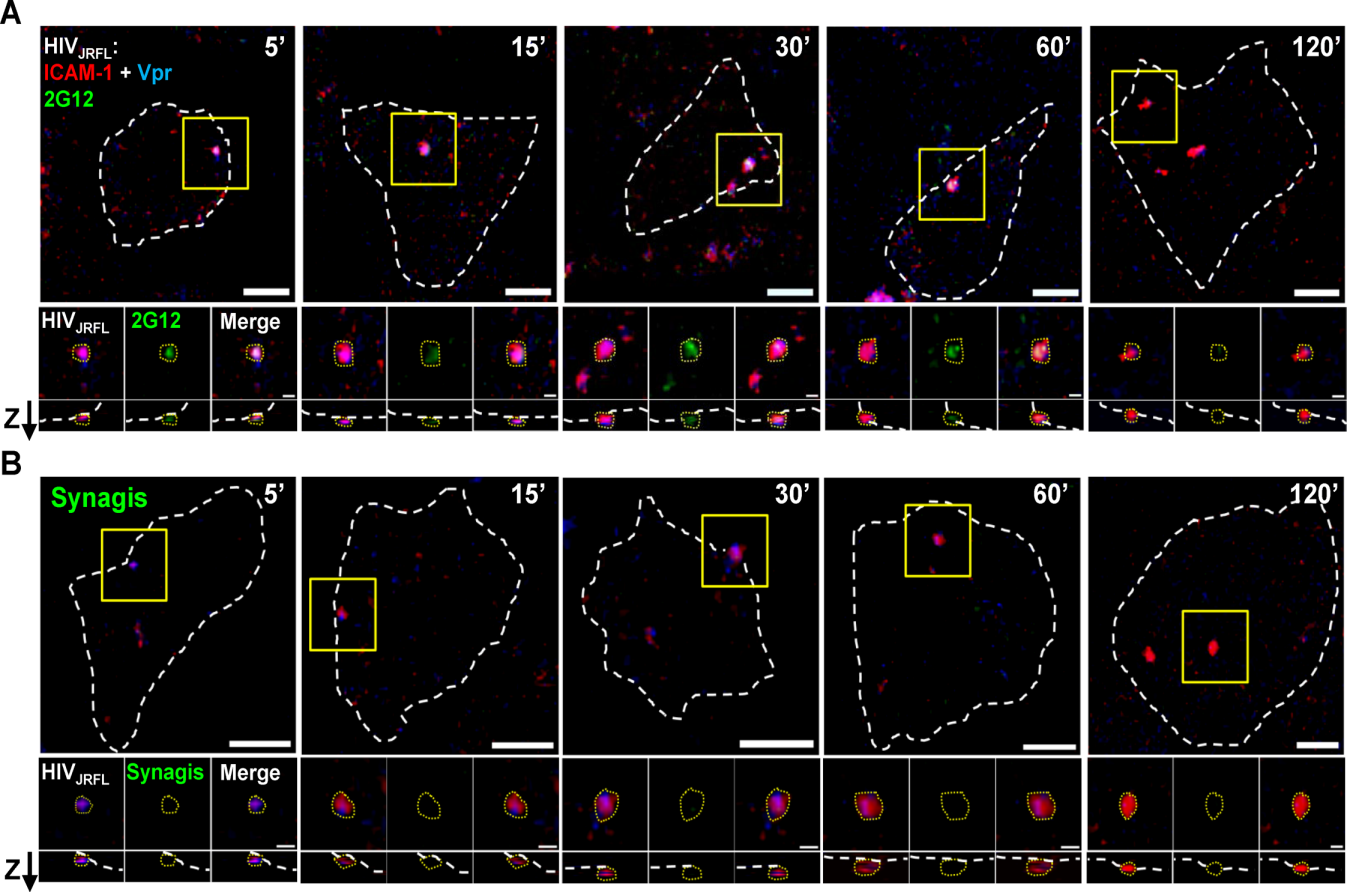
Representative fluorescence signals from cell-bound HIV_JRFL_ virions. HIV_JRFL_ pseudovirions expressing SNAP-ICAM-1 and CLIP-Vpr were fluorescently labeled with membrane-impermeable SNAP-Surface Alexa Fluor 546 (Red) and membrane-permeable CLIP-Cell Alexa Fluor 360 (Blue), respectively. Gp120 epitope exposure was probed at the indicated virus-cell contact periods with Alexa 488-tagged Mab 2G12 (A; green signal), versus identically labelled control Mab Synagis (B). Cortical actin detected by staining with Alexa-647-conjugated phalloidin is delineated by the white dashed lines (See S3 Fig). The top-most images in each panel show the entire surface of the cells in the lateral (XY) plane. Scale bar = 5μm. Yellow boxes mark magnified regions shown in the three images immediately below the corresponding top pictures. These images depict, from left to right, SNAP-ICAM-1 (red) plus CLIP-Vpr (blue) signals; antibody (green) signals; and merged signals. The lowest images show signals in the axial (Z) plane with the “Z” arrow pointing to the outer surface of the cell. The dashed yellow lines around virus particles represent example ROIs used to obtain fluorescence intensity (see Methods). Scale bar = 1μm.

Putative surface-bound virions were captured for analyses within regions of interest (ROI) that were defined as follows (also see Methods for details). First, the ROIs were selected based on the presence of a SNAP-ICAM-1 signal. Second, the ROI borders were configured to define where fluorescent signals fell to apparent background. Third, target cells were stained with phalloidin (which binds to cortical F-actin) in order to distinguish between the intracellular space and the cell surface. Only surface-bound particles, designated as such according to their orientation determined by phalloidin signals examined in lateral (XY) and axial (Z) orientations were considered for analysis. An example tracing of cell peripheries versus the virus—cell interface is shown in S3 Fig. For each selected ROI, a randomly selected mock ROI (i.e. one not containing an apparent fluorescent signal) was selected on a region of the same cell to define background fluorescence signals. All putative virion ROI fluorescence values were corrected for such background measures (see Methods). Fig. 2 shows representative images of ROI (selected using the criteria described above) in tests with either neutralizing Mab 2G12 (Fig. 2A) or Synagis negative control (Fig. 2B) conjugated to Alexa Fluor 488. As with free virions (Fig. 1), Mab 2G12 reacted with cell surface-bound virions starting within five minutes of virus-cell attachment (Fig. 2A). This was interpreted to indicate that envelope spikes on a subset of virions were oriented away from the cell surface and therefore free to engage antibody. No such reactivity was seen with Synagis (Fig. 2B).

### Different subpopulations of HIV-1_JRFL_ virions emerge over time post-attachment

A series of experiments were carried out to probe bound virions with Mabs b12, 2G12, A32, C11, 17b, and negative control Synagis. Labelled HIV-1_JRFL_ virions were bound to either TZM-bl cells or HeLa-CD4 cells for various periods of time up to 120 minutes at 37°C. All experiments were conducted under identical incubation conditions using the same preparation of labelled virus. To characterize the dynamics of HIV-1_JRFL_ bound to each type of target cell, we first examined SNAP-ICAM-1 and CLIP-Vpr fluorescence data obtained from at least 1800 ROIs per time point from across antibody experiments. The detection of Vpr by the membrane-permeable CLIP-Cell Alexa 360 dye was not attributable to the presence of degraded virions with exposed contents, as there was no staining of virions with anti-p24 antibody unless the particles were first treated to permeabilize viral membrane (representative images shown in S2A Fig).

**Fig 3:**
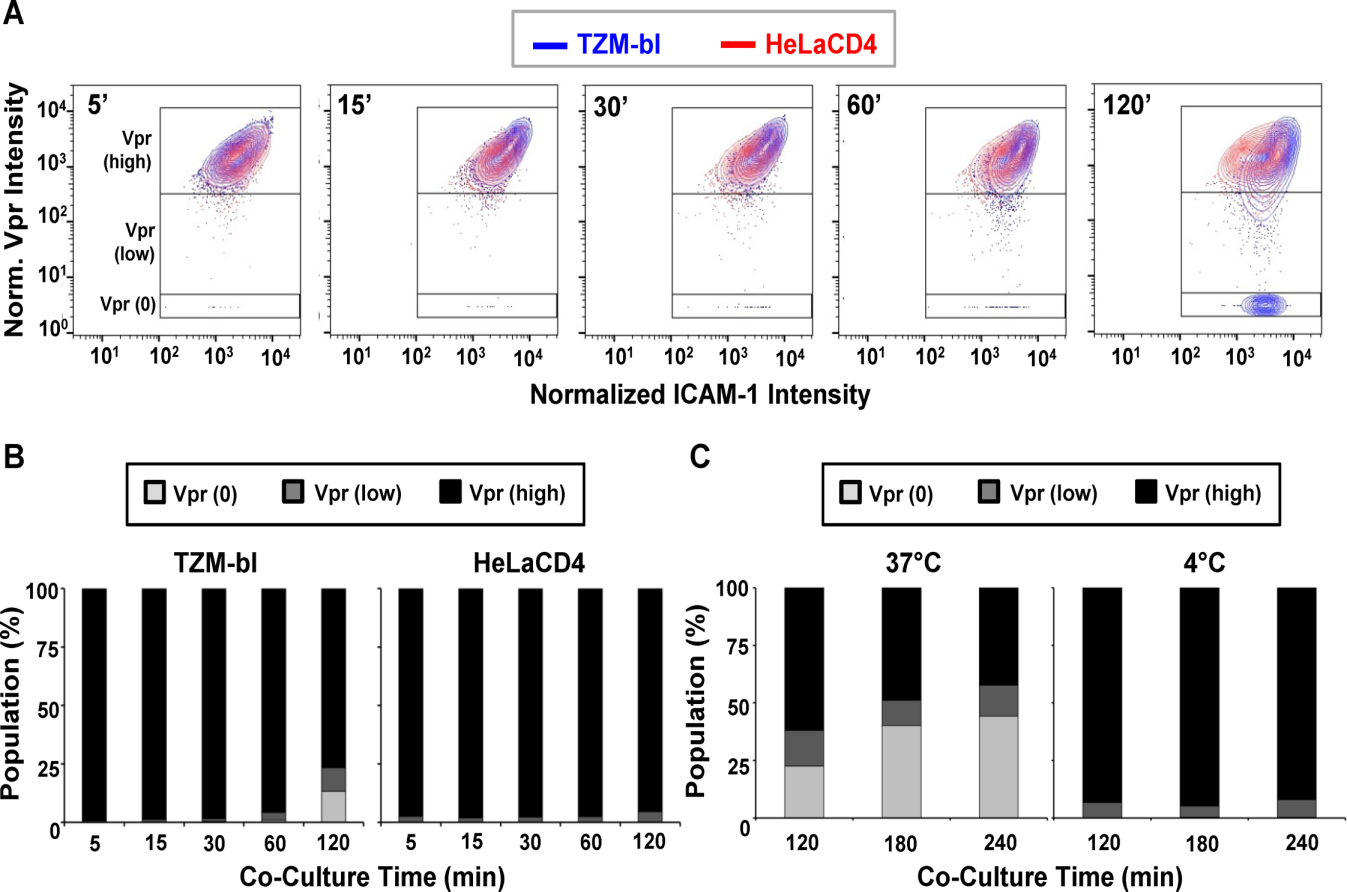
Changes in SNAP-ICAM-1 and CLIP-Vpr signals on HIV_JRFL_ pseudoviruses attached to TZM-bl or HeLa-CD4 target cells. (A) To characterize changes in HIV_JRFL_ during interactions with target cells, ROIs comprising cell-bound virus particles were selected and used to extract SNAP-ICAM-1 and CLIP-Vpr intensity signals. These data were then normalized in order to generate contour plots (see Methods) of HIV_JRFL_ signals on TZM-bl (blue) or HeLa-CD4 (red) target cells at the indicated times. Three Boolean gates were defined based on Vpr content at the 5 minute co-culture time point with TZM-bl cells: Vpr (0), corresponding to data points with no Vpr signal (bottom box); Vpr (low) corresponding to data in the bottom 5^th^ percentile of signal intensity (middle box); and Vpr (high) corresponding to the top 95^th^ percentile of the data set (top box). A minimum of 1800 ROI per condition are shown. (B) Based on the gating strategy described in (A), the Vpr (0) (light grey), Vpr (low) (dark grey), and Vpr (high) (black) subpopulations were quantified as percentages of the entire bound virus population on TZM-bl cells (left) or HeLa-CD4 cells (right) after the indicated times of attachment. (C) The same three subpopulations were quantified on TZM-bl cells after extended attachment periods (120–240 minutes) at the fusion permissive temperature 37°C (left) or at 4°C (right) where membrane mixing is arrested.

The raw intensity data was normalized, log-transformed, and then multiplied by 1000 for analysis using the FlowJo flow cytometry software as described in **Methods**. As shown in Fig. 3A, the selected virions reflected a range of SNAP-ICAM-1 and CLIP-Vpr signals on TZM-bl (Fig. 3A, blue) or HeLa-CD4 (Fig. 3A, red) target cells at five minutes post-attachment. The distribution of SNAP-ICAM-1 signal intensities fell within a 1.5 log range but was roughly the same on both target cell types and did not appreciably broaden or constrict over time in either case. In comparison, the CLIP-Vpr signals dispersed over more extended periods of time on the TZM-bl cells; in large measure because of the steadily increasing appearance of virion populations with low or no Vpr signal (Fig. 3A).

To more precisely examine the observed changes in CLIP-Vpr signals, Boolean gating analysis (using FlowJo) was used to partition virions into three populations representing high, low or no Vpr signal based on data for the 5-minute time point: Vpr (0), corresponding to data points with no Vpr signal; Vpr (low) corresponding to data in the bottom 5th percentile of signal intensity; and Vpr (high) corresponding to the upper 95th percentile of the data set. These gates were then transposed onto HIV_JRFL_ data sets at subsequent time points. Overall, the temporal analyses confirmed that ROIs circumscribing particles with low or no Vpr signal, which were rare early after cell binding, increased in frequency over time on the surfaces of TZM-bl cells (Fig. 3B). After 120 minutes of contact with TZM-bl cells, roughly 28% of virions had low or no Vpr signal. The fraction of surface-bound virions with high Vpr signal decreased reciprocally (Fig. 3B, Left panel). Changes in the Vpr (low) population were statistically significant for all adjacent time points (two-sample Kolmogorov-Smirnov test; p value corrected for multiple comparisons < 0.05) except for the 15 minute versus 30 minute times (S1 Table). On HeLa-CD4 cells, bound virions with a low Vpr signal remained relatively sparse (roughly 5 to 8%) and did not increase over time. Only a negligible amount of virions with no Vpr signal (<1%) was apparent at any time (Fig. 3B, right panel). Collectively, these data indicate that HIV-1 particles with low or no Vpr signal were very sparse in the initial virus preparation but evolved over time once bound to cell surfaces, in a cell type-specific manner.

We next asked whether the time-dependent appearance of ROIs with low or no Vpr signal on TZM-bl cells arises from protein-protein interactions or from virion-cell membrane intermixing. At low temperatures (i.e. less than 23°C), protein interactions can occur but membrane mixing cannot [42, 102, 103]. Accordingly, we examined the appearance and distribution of HIV-1_JRFL_ pseudovirus populations with high, low or no Vpr signals in the context of extended periods (up to 240 minutes) of interaction with TZM-bl cells maintained at 37°C or at 4°C. These experiments were also conducted using the same preparation of labelled virus as in the previous experiments.

As seen in Fig. 3C, at 37°C the fraction of ROIs with no Vpr signal increased over time on TZM-bl cells to reach roughly 40% of the total population examined. Notably, the fraction (11 to 15%) of bound particles with low Vpr signals remained relatively constant from 120–240 minutes at 37°C. In comparison, at 4°C, particles with no Vpr signal were very rare on TZM-bl cell surfaces (< 1% of the total population) even after prolonged incubation times. Particles with low Vpr signals were observed (5 to 7% of the total population) and did not change appreciably over time. Collectively, the data indicate that certain populations of cell-bound virions exhibit changes in content depending on time, temperature and target cell type.

### Gp120 epitope exposure exhibited by surface bound virions

We next evaluated gp120 epitope exposure on the virions bound to either TZM-bl or HeLa-CD4 cells as revealed by reactivity with b12, 2G12, A32, C11, and 17b anti-gp120 Mabs. Synagis was used as a negative control. At least 300 ROIs were defined per test antibody on virions bound to either target cell.

**Fig 4:**
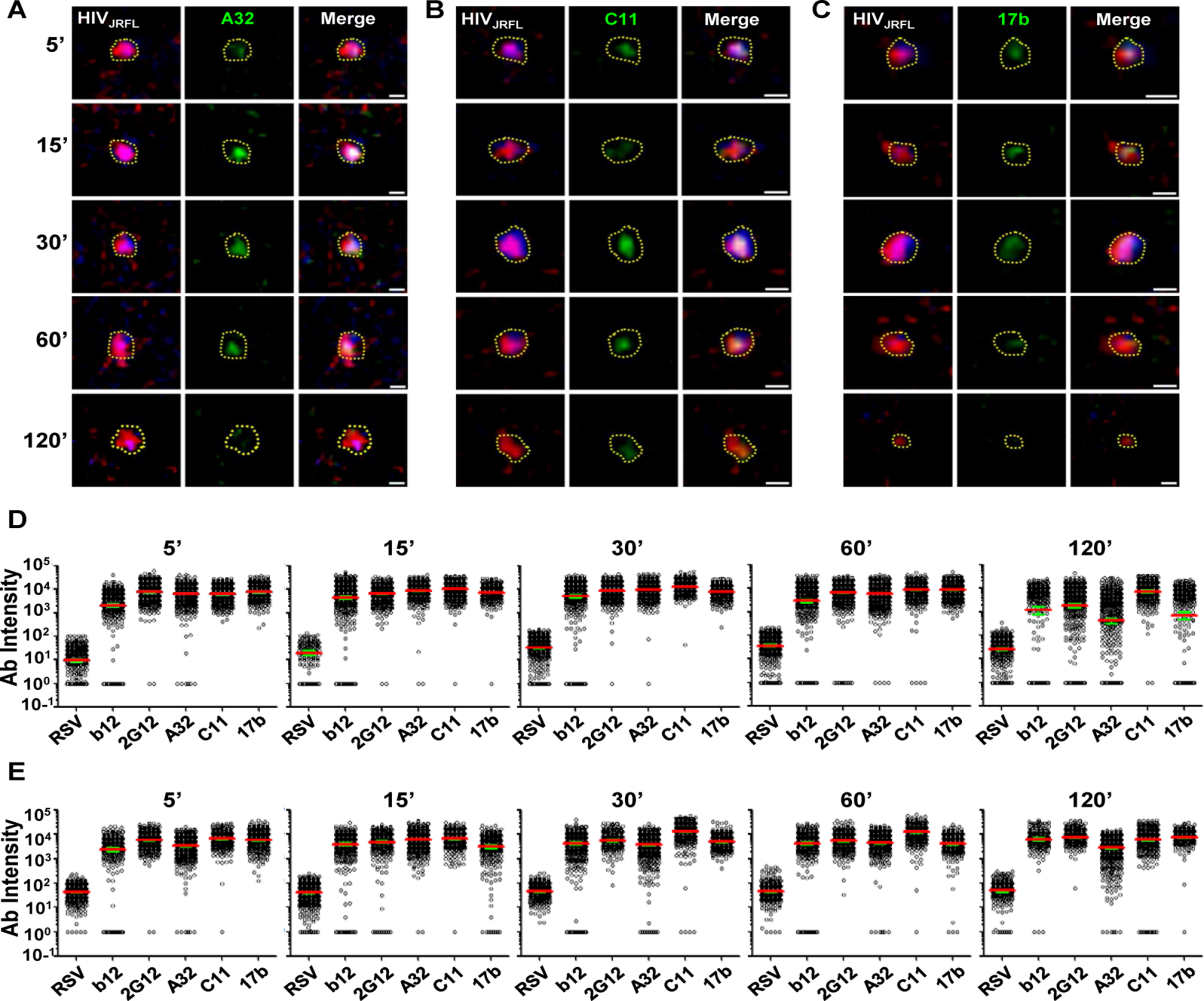
Gp120 epitope exposure on target cell-bound HIV_JRFL_ virions. Representative images of SNAP-ICAM-1 (Red) and CLIP-Vpr (Blue)-expressing HIV_JRFL_ virions bound to TZM-bl cells are shown. Virions were further stained with anti-CD4i Mabs (Green) A32 (A), C11 (B), or 17b (C) as described in Methods. Periods of virus-cell attachment are shown. Scale bar = 1μm. The dashed yellow lines mark example ROIs selected for fluorescence intensity measurements (see Methods). Relative anti-gp120 antibody intensity signals were compiled from ROIs on TZM-bl (D) cells or HeLa-CD4 cells (E) after the indicated attachment periods and graphed as arbitrary units of intensity (a.u.i). The test Mabs are indicated; Synagis served as negative control Mab. Each experimental condition contains at least 300 ROIs. Red lines represent the geometric mean of the data; green bars indicate standard errors.

Surprisingly, anti-CD4i Mabs A32 (Fig. 4A), 17b (Fig. 4B), and C11 (Fig. 4C) bound to attached virions in a manner similar to Mabs b12 and 2G12 (see Fig. 2). Fig. 4 shows a comprehensive perspective of Mab binding signals (expressed as arbitrary units of intensity; a.u.i) over time derived from ROIs on TZM-bl cells or HeLa-CD4 cells (panels D and E, respectively). The distribution of signals depended on target epitope, cell type and virus-cell interaction time. The broadest signal distributions were observed with Mabs 2G12, A32 and 17b signals on virions bound to TZM-bl cells for 120 minutes (Fig. 4D). Overall, the geometric mean signals from the negative control Mab Synagis were at least 1.5 logs lower than the values observed with any anti-gp120 Mab under all matched test conditions. These differences were significant (Kruskal-Wallis test; p < 0.0001).

As with virions attached to coverslips, free gp120 did not appear as a confounding factor on HIV-1_JRFL_ virions bound to target TZM-bl cells, as experiments with D7324 antibody were negative (representative images shown in S2B Fig). Further, virion degradation appeared to be minimal as there was also no anti-p24 staining within bound virion ROIs, including the Vpr (0) subpopulation (representative images shown in S2B Fig). As an additional control for nonspecific antibody binding patterns (i.e. ones that might occur in the absence of viral receptors), the tagged HIV-1_JRFL_ virions were co-cultured with parental HeLa cells (CD4- and CCR5-negative) for 30 minutes at 37°C, then fixed and stained with Alexa Fluor 488-conjugated test Mab as described above. As the washing step of the above procedure removed all virions from these cells, confocal analyses were performed on particles left to settle on cells at random. ROIs were defined as described above. Under these conditions, Mab signals were seen for the constitutively expressed epitope 2G12, but not with Mabs A32 and C11. There was marginal staining of some particles with CD4i Mab 17b (S4 Fig).

Next, we examined the exposure of gp120 epitopes after longer periods of virus interactions (120, 180, and 240 minutes) with TZM-bl cells at either 37°C, or 4°C. The cells were fixed and the attached virions stained with antibodies as in the previous experiments. At least 200 ROIs (defined as in the previous experiments) were surveyed for each experimental condition (S5 Fig). Under all conditions, the geometric mean fluorescence signal observed with Synagis did not change over time and was similar to what was observed during shorter time frames (see Fig. 4). The geometric mean fluorescence signals for all test Mabs was significantly lower on virions bound to TZM-bl cells at 37°C (S5 Fig, black) than at 4°C (S5 Fig, blue) (Kruskal-Wallis test: p < 0.0001). The difference in test Mab intensity at these two different temperatures increased with increasing co-culture time, from less than 1 log to approximately 2–3 logs. Most Mab staining levels were maintained at 4°C through the 240-minute incubation period, with the exception of Mab A32 where mean fluorescence signals dropped about 1 log between 180 and 240 minutes.

**Fig 5:**
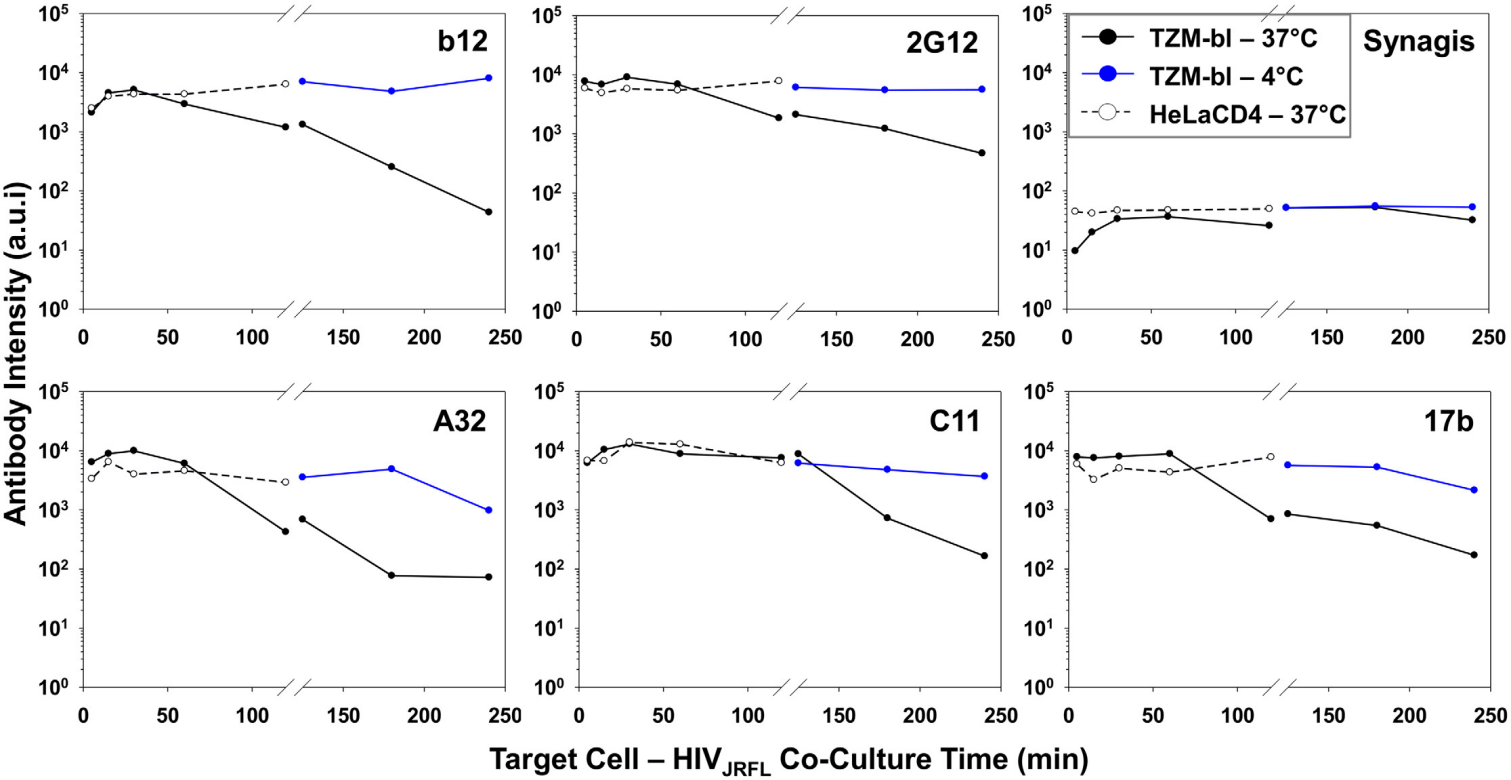
Influence of co-receptor expression, temperature and time on the exposure of gp120 epitopes on cell-bound HIV_JRFL_ virions. Geometric mean antibody intensity signals derived from the data in Fig. 4 and S5 Fig were compiled and plotted versus attachment time. Signals for particles attached to TZM-bl versus HeLa-CD4 cells from 5–120 minutes at 37°C are shown as black solid and dashed lines, respectively. Signals for particles attached to TZM-bl cells between 120 and 240 minutes at either 37°C or 4°C are shown as black or blue lines, respectively.

Fig. 5 summarizes the geometric mean fluorescence signals obtained from 5 to 120 minutes (shown in Fig. 4) of virus interaction with the two target cell types at 37°C, compared with signals measured on TZM-bl cells after longer time points (comprehensive data arrays shown in S5 Fig) at the two different temperatures. Overall, the compiled data show that temporal changes in antibody staining signals were dependent on assay conditions. Signals were relatively static on HeLa-CD4 cells at 37°C and on TZM-bl cells that were temperature arrested at 4°C, although in the latter case the Mab A32 and 17b signals declined by roughly one to one-half log between 120 and 240 minutes. The most extensive changes in Mab binding signal were seen on virions bound to TZM-bl cells (CD4^+^; CCR5^+^) at 37°C. Excepting Mab C11, fluorescence signals for the test Mabs began to decline after 60 minutes of virus attachment under these conditions. The most extensive changes were apparent with Mabs A32 and b12. Between 5 and 240 minutes there was a roughly 2–3 log drop in geometric mean fluorescence binding signals, reaching the background signal delineated by Synagis at the later time point. Mab C11 signals began to decline after 120 minutes of virus attachment to TZM-bl cells at 37°C and decreased roughly two logs by 240 minutes.

**Fig 6:**
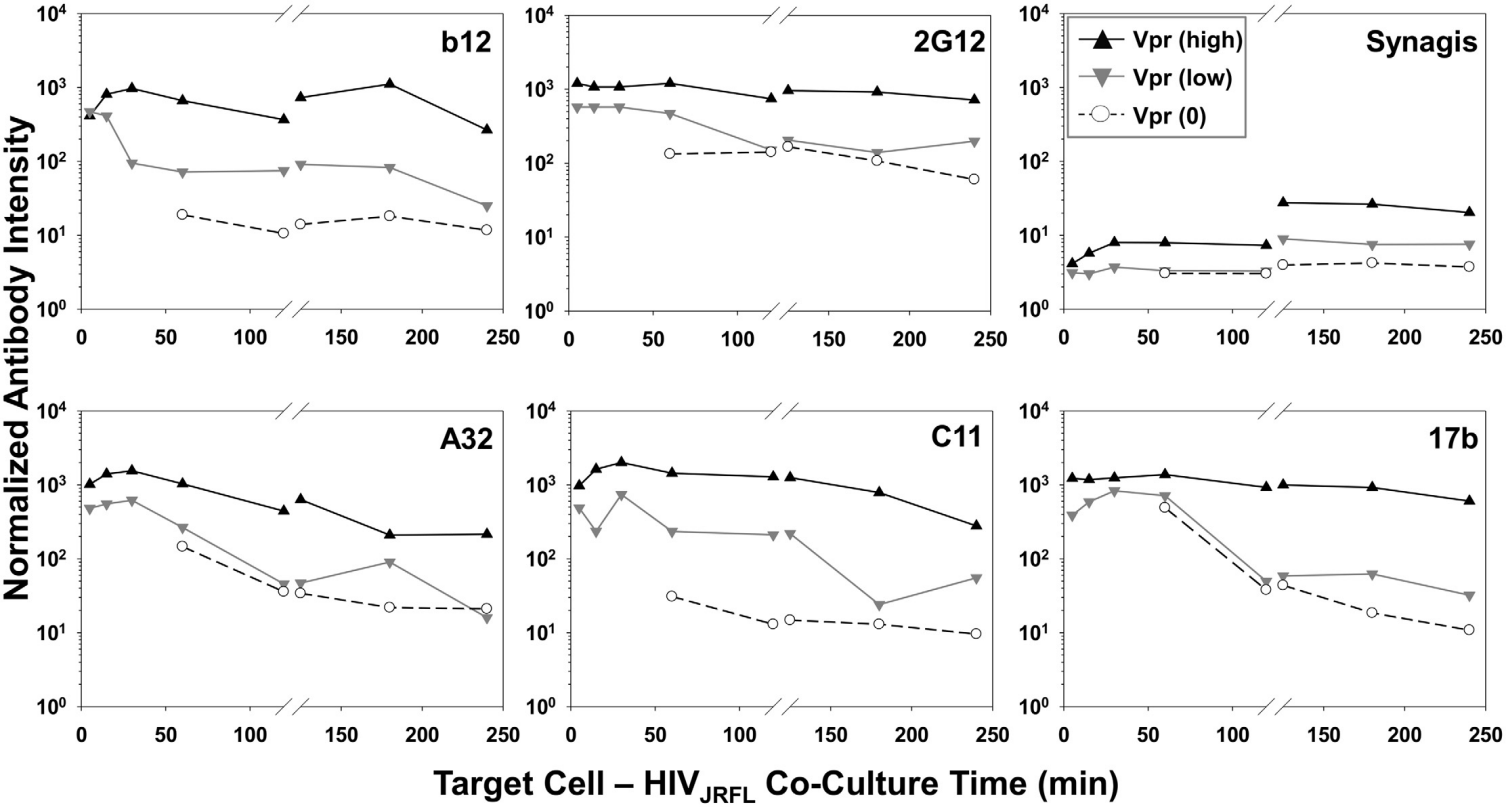
Exposure of gp120 epitopes on Vpr subpopulations of TZM-bl cell-bound HIV_JRFL_ virions. Normalized antibody intensities were determined for Vpr (0) (○ symbols), Vpr (low) (▼ grey symbols), and Vpr (high) (▲ black symbols) subpopulations of HIV_JRFL_ virions bound to cells at 37°C (See Fig. 3) using FlowJo software. Signals for 5–120 minute attachment times are derived from the same ROIs shown in Fig. 4, and signals for 120–240 minutes from ROIs in S5 Fig.

The temporal declines in Mab staining signals on TZM-bl cells under entry-permissive conditions of 37°C were explored in greater detail. In particular, we investigated whether this was associated with the gradual appearance of multiple cell-bound virion subpopulations that occurred most extensively under these conditions. To do this, Mab fluorescence signals were extracted from the Vpr (high), Vpr (low) and Vpr (0) subpopulations (see Fig. 3) using FlowJo software. For each Mab, mean fluorescence intensity signals observed within these subpopulations were then plotted over time (Fig. 6). The highest intensities for all test Mabs were recorded on the Vpr (high) subpopulation (Fig. 6). The lowest levels of Mab staining occurred on the Vpr (0) subpopulation that appeared after 60 minutes of virus- TZM-bl cell interactions (see Fig. 3). Mabs staining on the Vpr (low) populations on each cell type was intermediate and more dynamic. Mab b12 signals in this population decreased one-half log within 30 minutes of attachment then steadily declined to near background levels (defined by Synagis negative control measures) by 240 minutes. Mab 2G12 signals were more persistent, decreasing less than one-half log after 60 minutes and remained above background levels for 240 minutes. Signals from anti-CD4i epitope Mabs, A32, C11, and 17b dropped at least one to one-half log by 120 minutes; approaching background levels (Synagis equivalent levels) by 240 minutes of virus-cell attachment. Notably, the Vpr (0) population marginally reacted with Mab 2G12 at all the time points; even less reactivity was observed with Mabs b12 or C11. Somewhat higher binding signals in the Vpr (0) population were observed with Mabs A32 and 17b at 60 minutes of virus-cell attachment, but were lost with increasing times. Thus, it is likely that the general decline in Mab binding signals observed for the entire population of ROIs on TZM-bl target cells under entry-permissive conditions (see Fig. 5) could be explained in part by the temporal emergence of the low or no Vpr subpopulations.

### Three dimensional superresolution imaging of gp120 epitope exposure on TZM-bl-bound HIV-1_JRFL_ virions

Superresolution microscopy techniques can be used to study structural and functional aspects of HIV replication [104–112] at levels of resolution compatible with the size of a retroviral particle. Accordingly, to provide a more detailed validation of the confocal microscopy we analyzed the TZM-bl/HIV-1_JRFL_ system used here with three-color, three-dimensional direct stochastic optical reconstruction microscopy (3D dSTORM). This method provides a 10-fold increase in resolution (20nm lateral and ~50nm axial resolutions [113]) over standard confocal microscopy. The lateral resolution was determined by the average full width at half maximum (FWHM) measurements of all three fluorophores used. Neutralizing Mab 2G12 and anti-CD4i Mabs A32 and 17b were selected as epitope probes.

**Fig 7:**
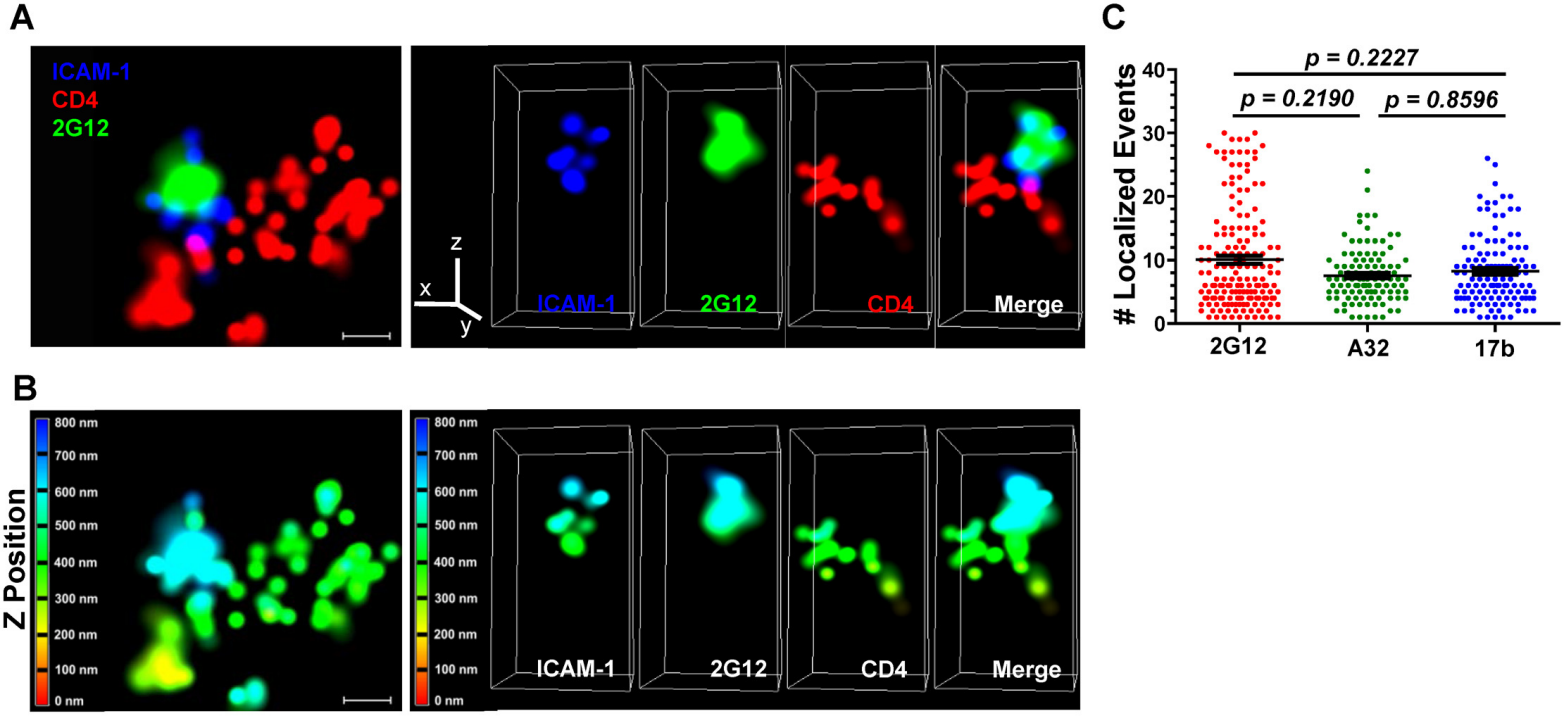
Superresolution imaging of Gp120 epitope exposure on TZM-bl-bound HIV_JRFL_ virions. HIV_JRFL_ virions were co-cultured with TZM-bl cells for 30 minutes and fixed. Gp120 epitope exposure was probed with Alexa 488-conjugated Mabs. (A) Top: A representative lateral dSTORM projection image of a virion bound to a TZM-bl cell. Cell surface CD4 is stained with Alexa 647-conjugated, anti-CD4 antibody OKT4 (red). The virion surface is marked by ICAM-1 tagged with membrane-impermeable SNAP-Alexa 546 (Blue); gp120 is stained by Alexa 488-conjugated Mab 2G12 (green). Scale bar = 0.1μm. Bottom: Image matches the one above, but with Z position scaling. The Z imaging range is divided into 100 nm sections; each is color coded as shown. Thus, signals with the same color fall within the same section (i.e. within 100nm). (B) Axial view of the dSTORM image in A, with the color channels separated as well as merged together. The Z line in the color channel panel is pointing upward away from the cell. The bottom images match the ones above, but with Z position scaling as in (A). Note that the ICAM signals are comprised within no more than two adjacent 100nm sections. (C) The number of localized events generated by Mabs 2G12, A32, or 17b in at least 110 superresolution ROIs containing bound HIV_JRFL_ virions is shown (see Methods). Black bars indicate the geometric mean and standard errors. The two-tailed Mann-Whitney test was used to perform pairwise comparisons of localized events measured with each Mab.

Fluorescently tagged HIV-1_JRFL_ virions (Alexa 546 labeled-SNAP-ICAM-1; Alexa 360-labeled CLIP-Vpr) were incubated with TZM-bl cells stained with a non-HIV neutralizing anti-CD4 antibody (Alexa 647-conjugated OKT4) for 30 minutes at 37°C. Co-cultures were then fixed with 4% paraformaldehyde, stained with Alexa Fluor 488-conjugated test Mab (2G12, A32, 17b or Synagis), and mounted in imaging buffer for examination using the Nikon N-STORM superresolution microscope (see Methods). The 3D dSTORM images were acquired and single molecule fitting and Gaussian images were rendered using the N-STORM software NIS Elements, where ROIs containing SNAP-ICAM-1and Mab signals proximal to CD4 staining were selected (representative images shown in Fig. 7 & S6 Fig; S1, S2, and S3 Movies). Consistent with the confocal microscopy results (Fig. 2, Fig. 4 & S4 Fig), neutralizing Mab 2G12 (Fig. 7, S1 Movie) and CD4i Mabs A32 (S6 Fig, S2 Movie) and 17b (S6 Fig, S3 Movie) produced a signal in proximity to SNAP-ICAM-1 and cell surface CD4. Importantly, scaling in the lateral plane versus the axial imaging range showed that SNAP-ICAM-1 and Mab signals fell within a 100–200 nm area, consistent with the size of a single retroviral particle (Fig. 7A, Fig. 7B, S6A Fig & S6B Fig, bottom panels). Overall, these data agree with the confocal imaging of ROIs in the TZM-bl/HIV-1_JRFL_ system, showing that both neutralizing and CD4i epitopes are found on bound particles.

Notably, the superresolution ROIs indicated that Alexa 488-conjugated Mabs 2G12, A32 and 17b generated comparable ranges of “blinking” fluorescence signals (localized events; Fig. 7C) when attached to cell-bound virions. In accordance, the Mabs shared consistent signaling characteristics when calibrated in the context of superresolution ROIs comprising a constrained gp120 antigen (see Methods and S7 Fig). The geometric mean of the localized events measured for Mab 2G12 was roughly two fold lower than the mean for Mabs A32 and 17b (S7 Fig, Panel A). There were no such differences between Mabs A32 and 17b. However, the distribution of localized events among ROIs was similar for all Mabs (S7 Fig, Panel B). ROIs with two localized events were most frequently detected with all antibodies; followed by ROIs with four localized events. ROIs containing larger numbers of localized events were relatively scarce (S7 Fig, Panel B).

## Discussion

Abundant evidence indicates that the susceptibility of HIV-1 to the antiviral effects of humoral immunity is dependent on how and when epitopes are expressed on the viral envelope glycoproteins. Intensive research has revealed a few sites of vulnerability on free virions, which comprise constitutively expressed epitopes with variable degrees of conservation among strains. On HIV-1 gp120, these include the CD4 binding site [23, 82–84, 91, 114] and glycan structures present on the variable loops [30, 61, 94, 115, 116]. More highly conserved regions including a variety of CD4i epitopes also exist on gp120 but appear not to be exposed on free virions [12, 51, 117–122].

Much less is known about the gp120 epitopes exposed on HIV-1 after attachment to target cell surfaces although the available evidence suggests that it differs substantially from that of free virions. Certain anti-envelope antibodies that bind poorly to free virions are capable of directly neutralizing infection, suggesting that exposure of neutralizing epitopes occurs after target cell attachment [24, 51, 63, 123]. More recently, we [55] demonstrated that virions bound to target cells present targets for Fc receptor-dependent antiviral mechanisms such as ADCC. Some of the more potent gp120 targets for such activity are located within the C1 region [55, 124, 125], which demands cell surface CD4 engagement for exposure [52, 60, 85, 86, 126, 127]. Additional targets include CD4i epitopes within the co-receptor binding site that also require cell binding for full exposure [16, 17, 51, 52, 55, 122, 128–130].

Such findings contrast with previous reports and *in silico* molecular models suggesting that CD4i epitopes are always occluded from immunoglobulin [43, 82–84] because of steric constraints at the cell surface. Importantly, this view of gp120 is derived largely from the crystal structures of soluble envelope glycoproteins and/or cryo EM images of engineered soluble trimers [131, 132] or free virions [22, 133–135]. However, such information may not fully reflect the antigenic profile of HIV-1 virions as they proceed through its attachment and entry steps.

HIV-1 epitope exposure on target cell surfaces has been successfully studied using confocal microscopy in systems where various reactants are fluorescently labeled. Such studies concerned epitope exposure during HIV-1 envelope-driven cell-cell fusion [16, 17, 136]; few studies have examined the disposition of surface bound particles [110, 137]. Standard confocal microscopy is incapable of the resolution needed to accurately position different fluorescence signals on single retroviral particles. Even under ideal conditions (e.g., a high numerical aperture and optimized laser alignment) the resolution of standard confocal microscopy is limited to approximately half of the wavelength of the excitation laser, the shortest of which (200 – 250nm) is larger than the size of an HIV-1 particle (0.145–0.181 μm) as indicated by high-resolution methods including electron microscopy or optical trapping [138, 139]. Nevertheless, standard confocal microscopy can be adapted to derive multiple fluorescence measures emanating from ROIs comprising bound virions. Furthermore, confocal microscopy is sufficiently robust to capture temporal information from populations of bound virions in order to reveal changes in the dispositions of HIV-1 components versus surface epitopes [16, 17, 137].

In the present study, we explored this question using virions expressing surface (SNAP-ICAM-1) and internal (CLIP-Vpr) fluorescent tags that can also be stained with Mabs conjugated to harmonious fluorescent labels (see Methods). Multi-parameter fluorescence data for populations of virions imaged over time was then normalized for Boolean gating in order to reveal concurrent changes in the dispositions of proteins expressing the various fluorescent signals.

HIV-1 has been reported to engage in endosomal entry [14, 15, 140–145] and/or endosomal recycling [146] in addition to direct virus-cell membrane fusion at the cell surface. A recent study by Herold et al. [147] showed that productive HIV-1 entry occurs predominantly at the plasma membrane, and does not require endocytosis. We focused exclusively on surface-bound virions since they are the most plausible targets for various humoral anti-HIV-1 effector mechanisms and/or for other antiretroviral agents designed to block viral entry. Further, the antigenic profiles of internalized virions are likely to be clouded by overlapping processes of endosomal versus lysosomal uptake [143] potentially involving productive infection or virion degradation, respectively. Accordingly, ROIs were selected for collection of fluorescence information based on outer membrane surface orientation along with a calibrated size corresponding to the apparent size of a retroviral particle. Although particles engaged in endosome-related processes may have been present in the system used here, they would not have been captured in ROIs since cell permeabilization occurred after anti-gp120 Mab treatment, surface fixing, and washing. ROIs without Mab signals were considered only if their orientation was on the extracellular surface. Three dimensional superresolution imaging showed that ROIs on the cell surface were proximal to CD4 and contained fluorescent signals comprising the dimensions of a retroviral particle (Fig. 7 and S6 Fig). However, we cannot completely eliminate the possibility that some of the ROIs defined by confocal imaging (e.g., those with more irregular contours) occasionally contained two or three HIV-1 particles captured in close proximity by the target cells.

The overarching immunological feature indicated by our analyses was that virions bound to target cells rapidly expressed an array of conserved gp120 epitopes including ones (e.g., A32, 17b, and C11) that were not exposed on the same virions when probed in solution (Fig. 1). Further, the signals obtained with Mabs specific for these epitopes were generally on par with what was observed with Mabs against constitutively exposed epitopes such as b12 and 2G12 (Fig. 2, Fig. 4, and Fig. 7). The exposure of the 2G12 epitope in our experiments agrees with previously published superresolution microscopic studies [110] showing that this epitope is expressed on surface bound virions. In this context exposure of the b12 epitope, which forms part of the CD4 binding site on gp120, is most likely attributable to the presence of epitopes on the virion face oriented away from the target cell.

All gp120 epitopes examined here exhibited a time-dependent reduction in immunoreactivity with cognate antibody (Fig. 4, Fig. 5, S5 Fig). However, such changes varied among the entire population of bound virions and were linked to conditions permissive for downstream membrane fusion and entry events. This was evident from findings that epitope exposure patterns were relatively static on HeLa-CD4 cells not expressing co-receptor or on TZM-bl cells held at low temperatures that prohibit membrane mixing (Fig. 5, Fig. 6, and S5 Fig). Based on these data, we posit that the broad decrease in gp120 epitope immunoreactivity seen on entry-permissive cells is linked to the presence of CCR5 co-receptor and downstream replication steps including the entry process itself. Possible mechanisms include the occlusion of epitopes via the repositioning of envelope spikes on attached virions, as was indicated by cryo-electron microscopy [134, 148]. An alternative albeit more speculative explanation is that the fully “opened” structure of CD4- and co-receptor-bound gp120 is uniquely susceptible to proteolytic degradation. Since our analyses were deliberately focused on extracellular processes, such degradation would have to occur at the outer cell surface to explain our findings.

In this regard, it was intriguing that CLIP-Vpr signals declined and/or disappeared over time within subsets of particles attached to entry-permissive TZM-bl cells (Fig. 3). The expansion of these subsets occurred after 30 minutes of virus-cell incubation at 37°C, which is consistent with previous observations of a similar “lag time” before transition state envelope structures and evidence of membrane fusion are detected [16, 17, 137, 149, 150]. Importantly, such particles were very rare at early time points and therefore were not dominant artifacts in the virus preparations used for our experiments. Further, the population with no detectable CLIP-Vpr signal appeared only on the TZM-bl cells expressing both CD4 and CCR5 and not on the CCR5 negative HeLa-CD4 cells (Fig. 3). Conversely, this population did not appear on TZM-bl cells at 4°C, where membrane fusion was temperature-arrested. Only a fraction of the particles bound to TZM-bl cells at high temperature acquired the low or no Vpr signal profile over time (Fig. 3C). Nevertheless, the occurrence of these particles is consistent with conventional models of virion fusion with the plasma membrane [151–160]. In this case, receptor-driven membrane mixing processes are predicted to release virion content into the target cell thus generating particles with low or no Vpr signal. However, loss of Vpr at the cell surface is at odds with models require endosomal uptake during or after CD4 and co-receptor engagement [13, 15] for fusion, entry and infection. These models would predict that Vpr should be retained within all particles that rest on the target cell surface. Our data are consistent with another proposed scenario [13, 150] in which some particles progress beyond hemifusion at the plasma membrane to create small pores which release a limited amount of virion content. Vpr is a plausible component of such release if a fraction of the protein is located beneath the viral envelope as has been reported [161, 162]. The transducing properties of Vpr might further promote movement out of the virion and into the cell [163]. An alternative possibility is that content release occurs when virions contact adjacent surfaces of the cell; e.g., as might occur between tightly spaced microvilli [164–166]. In this situation, lateral forces could enable a process of “fusion from without” [13, 167]. Finally, our observations might reflect processes that lead to the specific loss of Vpr signal. Exploration of such questions will require future studies that simultaneously track multiple intra-virion components.

Another noteworthy feature of the bound particles with low or no Vpr signals was that they poorly expressed the gp120 epitopes examined here (Fig. 6). Thus, the processes on TZM-bl cells that led to the temporal decline in CLIP-Vpr signals may have concurrently impacted the disposition of the viral envelope. The broad loss of epitope reactivity with antibody is consistent with either the extensive degradation or loss of gp120 as has been studied previously [168–173]. Alternately, in view of the potential mechanisms for CLIP-Vpr signal reductions discussed above, epitope loss may occur as part of an active or abortive entry processes. It must be noted that certain features of epitope exposure were less apparent in the entire population of bound particles (Fig. 5) compared to what was seen in subpopulations (Fig. 6). For example, within the entire HIV_JRFL_ virion population (Fig. 5) epitope reactivity with anti-gp120 antibodies declined with increasing attachment time on TZM-bl cells. Examinations of subpopulations (Fig. 6) revealed that Mab staining was retained on Vpr (high) but progressively lost on Vpr (low) or (0) particles (Fig. 6). Precise links between SNAP-ICAM-1, CLIP-Vpr and Mab signal patterns are difficult to establish with population-based studies such as this one. However, live-cell imaging of fluorescent signals from single particles in real time may reconcile such questions. Data from the current study provide a foundation for such future efforts.

Emerging techniques of superresolution microscopy have been increasingly applied toward studies of HIV replication [104–112]. The application of one such technique; three dimensional, three-color dSTORM, allowed us to obtain a more refined view of the TZM-bl cell-bound HIV_JRFL_ virions examined in this study. This approach revealed that ROIs such as those selected for confocal microscopy comprised SNAP ICAM-1-and anti-gp120 Mab fluorescence signals that were co-localized within an area less than 200nm (Fig. 7 & S6 Fig) in size, consistent with measures of HIV virions by cryo-electron microscopy [138]. Further, these signals occurred within biologically relevant proximity to signals from anti-CD4 antibody bound to cell surface CD4. Mabs 2G12, A32 and 17b produced such co-localized signal patterns, in agreement with the fluorescence signals measured by confocal analyses (Fig. 2 & Fig. 4) of cell-bound virions.

An interesting question concerned how many gp120 epitopes on a cell-bound virion might react with cognate Mabs under these conditions. This was approached by first calibrating the number of localized events produced by each of the anti-gp120 test Mabs when bound to a delimited amount of known target antigen. The methods we used (see Methods and S7 Fig) were deliberately conformed to preserve intact antibody interactions with native gp120 structures. The latter feature was accomplished via D7324 antibody capture of a single chain gp120-CD4 complex (FLSC) that presents a stabilized CD4-induced structure [174]. A caveat was that such ROIs could variably contain one or two target antigens captured by a D7324 antibody. Thus, the total localized events measured could derive from the formation of either one or two immune complexes with the conjugated anti-gp120 Mabs. However, this possibility applied equally to all Mabs tested. In the calibration system, Mab 2G12 trended toward slightly fewer localized events compared to Mabs A32 and 17b although in all cases ROIs with two localization events were most frequently observed (S7 Fig). In comparison, the differences between numbers of localized events generated by the Mabs on TZM-bl cell-bound virions was not significant (Fig. 7C), in accordance with the similar signal intensity levels detected with the antibodies in confocal microscopy (Fig. 4D). Accounting for these considerations, we could estimate that TZM-bl cell-bound virions reacted with roughly 5–10 Mab 2G12 molecules; 3–6 Mab A32 molecules; or 4–8 Mab 17b molecules. In agreement with these estimates, previous superresolution microscopy studies using Stimulated Emission Depletion (STED) [110], indicated that on average Mab 2G12 bound to 7 trimeric spikes on a mature HIV virion. Other published studies using protein purification [175] and electron tomography [176] suggest that there are between 7 and 14, or from 8 to 10 spikes per HIV-1 particle, respectively.

Taken together, the patterns of gp120 epitope exposure revealed by imaging of cell-bound HIV-1_JRFL_ virions are inconsistent with models in which CD4i epitopes are predicted to be fully occluded from antibodies at the cell surface [43, 82, 84]. There are two possible explanations for this disagreement. The simplest one is that the CD4i and other epitopes are not buried at interfaces where virions contact target cell membranes to the extent that interactions with immunoglobulins are prevented. The second, more speculative possibility is that HIV-1 binding to cell surface receptors propagates conformational changes across the virion that impact envelope spikes distal to the cell contact zone. Such plasticity has been observed with other enveloped viruses [177–183].

A related question concerns why antibodies to CD4i epitopes such as A32 are not directly neutralizing even though the cognate epitopes are exposed at the cell surface. The exposure of such epitopes without direct neutralizing consequences was previously established for HIV-driven cell-cell fusion [16, 17]. Likely explanations pertinent to cell-bound virions include: epitope exposure occurs on trimers that fail to fully enable membrane fusion machinery; epitope exposure that occurs after the membrane fusion process has been committed; epitope exposure that occurs distal to the cell contact zone as suggested above. Further exploration of these possibilities in the context of HIV-1 attachment will require more advanced spatial analyses using superresolution microscopy and other molecular techniques.

Importantly, the gp120 imaging patterns elucidated here for attached virions are entirely consistent with previous findings that certain anti-CD4i epitope Mabs mediate potent ADCC activity in vitro against virions bound to target cells [55]. This accordance supports the concept that there may be multiple opportunities for humoral responses to counter HIV-1 infection after attachment. Following the observations made here, it could be envisioned how Fc receptor-dependent modes of humoral immunity might locate and destroy cells recently targeted by HIV-1 for replication. In this case, the ensuing effector cell activity might have a protective effect even if it is directed toward replication defective particles if other virions on the same cell manage to initiate productive replication. However, the efficacy of such responses in vivo is obviously dependent on the number of virions that attach to any given target cell, the durability of epitope exposure during initial stages of HIV-1 replication, and the proximity of effector cells to the epicenter of HIV-1 replication. Our data suggest that gp120 epitope exposure on attached virions is transient but sustained for periods of time that might allow immune mechanisms to impact infection under certain conditions. Information from clinical trials of candidate HIV-1 vaccines along with systematic testing of anti-gp120 Mabs in various animal models of HIV-1 infection could help to reconcile this question.

## Methods

### Cells

HeLa cells, which express CXCR4 but not CD4 or CCR5 (CD4-, CCR5-) [7, 8, 184], HeLa-CD4 clones (CD4+, CCR5-) stably transfected to express CD4, and TZM-bl cells expressing CD4 and CCR5 (CD4+, CCR5+) were used. TZM-bl cells were obtained through the NIH AIDS Research and Reference Reagent Program, Division of AIDS, NIAID, NIH: TZM-bl from Dr. John C. Kappes, Dr. Xiaoyun Wu and Tranzyme Inc. [98–101]. The HeLa-CD4-LTR-β-gal cell line with high CD4 expression was also obtained through the AIDS Research and Reference Reagent Program, Division of AIDS, NIAID [185]. Both cell lines were maintained in Dulbecco modified Eagle medium (DMEM; Gibco-BRL) supplemented with 10% heat-inactivated fetal bovine serum (FBS), 2 mM L-glutamine, and antibiotics, with 0.1 mg of G418 (Gibco-BRL)/ml, and 0.05 mg of hygromycin B/ml supplements for HeLa-CD4 cells in a 37°C incubator with 5% CO_2_. CD4 and CCR5 content of these cells was quantified using the QuantiBRITE PE fluorescence quantitation kit (BD Biosciences). TZM-bl cells had 1.7 x 10^5^ CD4 and 5.4 x 10^4^ CCR5 molecules/cell, while HeLa-CD4 cells had 1.8 x 10^4^ CD4 molecules/cell and no CCR5.

### Plasmid Constructs

To make pSNAP-ICAM-1, human ICAM-1 was extracted from the pCDM8-ICAM-1 vector (Addgene, Cambridge, MA) using PCR, and cloned into the SNAP-tag expressing pSEMXT-26m plasmid (New England Biolabs, Ipswich, MA) at the 3’ end of the SNAP-tag coding region using the SbfI and BamHI restriction sites. To make pCLIP-Vpr, the Vpr coding region was extracted from pEGFP-Vpr vector obtained through the NIH AIDS Research and Reference Reagent Program, Division of AIDS, NIAID [186] by PCR, and cloned into the pCLIPm vector (New England Biolabs) at the 3’ end of the CLIP-tag coding region using the SbfI and BamHI restriction sites. pSNAP-ICAM-1 and pCLIP-Vpr were sequenced by the Biopolymer Laboratory of the University of Maryland School of Medicine and assigned GenBank accession numbers Banklt1758508 Seq1 KM555100 and Banklt1758535 Seq1 KM555101, respectively.

### HIV-1_JRFL_ Pseudovirus Production

CCR5-tropic HIV-1_JRFL_ pseudoviruses were generated by co-transfecting HEK 293T cells with (i) pSG-3ΔEnv virus backbone with an Env deletion obtained through the AIDS Research and Reference Reagent Program, Division of AIDS, NIAID [187, 188]], (ii) pCAGGS-JRFL, a plasmid containing JRFL Env, obtained through the AIDS Research and Reference Reagent Program, Division of AIDS, NIAID [189] (iii) pSEMXT-ICAM-1 (expresses SNAP-tagged ICAM-1 captured by virions from the cell membrane during viral budding [190, 191], and (iv) pCLIP-Vpr (expresses CLIP-tagged Vpr marking virion content) [157–160]. Transfections were accomplished using FuGENE 6 (Roche, Indianapolis, IN) transfection reagent at a 3:1 reagent-DNA ratio. Pseudovirus-containing supernatant was harvested after 3 days, and concentrated about 10-fold by incubating with PEG-*it* virus precipitation solution (System Biosciences, Mountain View, CA) for 18 hours at 4°C as recommended by vendor. The antigen content of pseudoviruses was quantified using p24 and gp120 ELISAs, and their TCID50 was obtained as previously described by Li et al. [192]. HIV-1_JRFL_ pseudoviruses with gp120 to p24 ratio of 1:10–1:50, and 200,000–500,000 TCID50/mL (FCS) or 1 x 10^6^–3 x 10^6^ TCID50/mL (Confocal and Superresolution microscopy) were used.

### Antibodies

We probed anti-Env epitope exposure on free HIV-1_JRFL_ as well as on cell-attached virions by examining the binding properties of fluorescently-labeled cognate human monoclonal antibodies (Mabs). 2G12 mAb was purchased from Polymun Scientific (Vienna, Austria); b12, A32, C11 and 17b, were expressed from plasmid clones using an IgG1 backbone for heavy-chain variable regions and either a κ- or λ-chain expression vector for light-chain variable regions by transfecting HEK 293T cells. Mabs were purified from culture supernatants by protein-A chromatography. A32 and 17b were initially provided by James Robinson, Tulane University, New Orleans, La. The humanized monoclonal anti-respiratory syncytial virus (RSV) antibody, SYNAGIS® (MedImmune LLC, Gaithersburg, MD), and human plasma IgG (Calbiochem, La Jolla, CA) were used as non-specific negative controls. D7324 (Aalto BioReagents, Dublin, Ireland), an antibody that binds the C terminus of monomeric gp120, and an HIV-1 p24 antibody (Abcam Ab9071, Cambridge, MA), were used to assess gp120 dissociation from HIV-1_JRFL_ trimers and exposure of capsid, respectively. OKT4, an antibody against CD4 that does not neutralize HIV-1, was purchased from BioLegend (San Diego, CA).

These Mabs were fluorescently labeled with Alexa Fluor 647 (for FCS experiments) and Alexa Fluor 488 (for confocal and superresolution microscopy experiments) using monoclonal antibody labeling kits from Molecular Probes (Eugene, OR) following manufacturer instructions. Briefly, 100 μg of Mabs were labeled with Alexa Fluor reactive dyes which have a succinimidyl ester moiety that reacts efficiently with their primary amines and form stable dye-protein conjugates. The labeled Mabs were separated from unreacted dye by centrifugation through a spin column at 1100 x g. The dye:protein ratio of the recovered Mabs was measured using a UV-vis spectrometer (Nanodrop 2000, Thermo-Scientific). Conjugated Mabs used in our experiments had an optimal ratio range of 4–6 (Alexa Fluor 488) or 3–5 (Alexa Fluor 647) moles of dye per mole of Ab.

### Fluorescence Correlation Spectroscopy

Fluorescence Correlation Spectroscopy (FCS) is a methodology that allows real-time detection of protein-protein interactions in solution, by measuring diffusion and reaction kinetics of fluorescently-labeled biomolecules [193]. The binding of Alexa Fluor® 647-labeled Mabs b12, 2G12, A32, C11, 17b, and anti-RSV antibody, Synagis (negative control) to HIV-1_JRFL_ was monitored by tracking diffusion of their fluorescent label across the observation area, where unbound antibodies will diffuse much faster than those that bound viral particles as described in [51]. Briefly, HIV-1_JRFL_ pseudovirions were diluted to 10μg/mL p24 equivalent in a 100-μL reaction volume (gp120:p24 ratio of 1:50), and were first incubated with 100μg/mL non-specific IgG1 (1.5μL of a 7mg/mL stock) for 90 minutes at 37°C to block non-specific binding. Then 1μL of the test Alexa Fluor® 647-conjugated Mab (4.5–6.6μg/mL) was introduced and allowed to interact with pseudovirions for 90 minute at 37°C. In experiments where CD4-induced conformational changes were studied, HIV-1_JRFL_ pseudovirions were pre-incubated with 100μg/mL sCD4 (Biogen) (1.5μL of a 7mg/mL stock) along with the non-specific IgG1 for 90minutes at 37°C before the addition of test Alexa Fluor 647 Mab. For spectroscopic measurements, 11μL of the reaction mixture was loaded onto an FCS slide reservoir, sealed, then placed on the Picoquant MicroTime 200, a time-resolved confocal microscope (inverted), with a high numerical aperture (NA = 1.3) oil objective (100× magnification). The samples were excited with *λ*ex = 635 nm laser, and fluorescence signals from the Alexa Fluor 647 Mabs were collected over 60 seconds in a constant detection volume that is continuously replenished. PicoQuant Symphotime software was used to generate the autocorrelation function of the fluorescent fluctuations of the Alexa Fluor® 647 Mab signal. The autocorrelation function of fluorescence intensities is given by the product of the Mab intensity at time *t*, *I(t)* with the intensity after a delay time *τ*, *I(t+τ)*, typically in the range from 10^-2^ to 10^2^ ms, averaged over the 60 seconds of measurement. For experiments where only the Alexa Fluor 647- Mabs were in the sample, the autocorrelation function was fitted with the pure diffusion model equation for single species, where a diffusion coefficient of 65μm^2^/sec was extracted for the 150kD IgG molecules. In the presence of HIV-1_JRFL_ pseudovirions, the autocorrelation was fit to a two-species diffusion model, where one species had the unbound Mab diffusion coefficient of 65μm^2^/sec (*D*_*nb*_), and the second species with a diffusion coefficient of 8μm^2^/sec (*D*_*b*_), represented Mabs bound to the 100nm HIV-1_JRFL_. These equations were also used to obtain the amount of Mab exhibiting the slower diffusion rate as a measure of the percentage of virus-bound Mabs in each reaction mixture. The derivation of all the equations is described in [51].

### Confocal Microscopy

4.0 x 10^5^ cells (HeLa, TZM-bl, or HeLa-CD4) were attached to 22-mm^2^ glass coverslips (Fisher Scientific) and incubated overnight at 37°C in 5% CO_2_. The next day, HIV-1_JRFL_ pseudovirions expressing SNAP-ICAM-1 and CLIP-Vpr were fluorescently labeled with their respective substrates, membrane-impermeable SNAP-Surface Alexa 546 (Red, Ex_max_: 558nm, Em_max_: 574nm), and membrane-permeable CLIP-Cell Alexa Fluor 360 (Blue, Ex_max_: 357nm, Em_max_: 437nm) (New England Biolabs), by incubation for 20 minutes at 37°C. TZM-bl and HeLa-CD4 cells grown on coverslips were co-cultured with 0.5mL of 1 x 10^6^–3 x 10^6^ TCID50/mL (equivalent to 7.0 x 10^5^–2.1 x 10^6^ PFU; ~ 1–3 MOI) of these fluorescently-labeled HIV-1_JRFL_ pseudovirions for the indicated times (5, 15, 30, 60, 120, 180, or 240 minutes) at either 37°C or 4°C, washed, then immediately fixed with 4% Paraformaldehyde (Electron Microscopy Sciences, Hatfield, PA) for 15 minutes. Experiments with receptor-negative parental HeLa cells eliminated the wash step, as this operation removed all viral particles (due to the absence of cell surface receptors). Thus, for microscopy the particles were left settled on the cells. Non-specific interactions were blocked by incubating coverslips with 10% normal goat serum (Thermo Scientific, Rockford, IL) and 100μg/mL non-specific IgG1 solution for 30 minutes at 4°C, then the specific epitopes were recognized by incubating cells with 5μg/mL Alexa Fluor 488 (Green, Ex_max_: 495nm, Em_max_: 519nm)-conjugated Mabs (b12, 2G12, A32, C11, 17b, or Synagis) for 1 hour at 4°C. For actin staining, after the addition of Mabs, coverslips were post-fixed with 4% Paraformaldehyde for 10 minutes, permeabilized in 0.2% Triton X-100 (Sigma) then incubated with Alexa Fluor 647-conjugated Phalloidin (Invitrogen) for 30 minutes at room temperature. Coverslips were mounted in Fluoromount (Sigma) and observed using the Zeiss Laser Scanning Microscope (LSM) 5 DUO. To verify that our results were independent of the fluorophore choices, we alternatively labeled HIV-1_JRFL_ with SNAP-Surface Alexa 546 (Red, Ex_max_: 558nm, Em_max_: 574nm) and CLIP-Cell BG505 (Green, Ex_max_: 505nm, Em_max_: 532nm), and recognized anti-Env epitopes with cognate Mabs conjugated with Alexa Fluor 350 (Blue, Ex_max_: 346nm, Em_max_: 442nm).

To view unbound virions by microscopy, 22-mm^2^ glass coverslips were coated with 0.1% Poly-L-Lysine w/v in water (Sigma) overnight at room temperature. The Poly-L-Lysine was removed and coverslips were washed with 1X PBS. Fluorescent-tagged HIV-1_JRFL_ pseudovirions (SNAP-Surface Alexa 546 and CLIP-Cell Alexa Fluor 360) were added and incubated for 2 hours at 4°C. Unbound virions were then washed off, and virions were fixed in 4% paraformaldehyde, non-specific interactions blocked with 10% normal goat serum, and test Alexa Fluor 488-conjugated Mabs added as described above.

### Confocal Microscopy Image Acquisition and Analysis

Virus—cell interactions were imaged using the Zeiss Laser Scanning Microscope (LSM) 5 DUO using a 63X oil-immersion lens at room temperature, where signals from the dually labeled virus and anti-HIV-1 antibody signals were tracked in 3D by taking 512 by 512 XY scans, 0.279018 μm/pixel size, in 0.2-μm increments in the Z-direction. Images were taken at 5 or more randomly selected regions that span the coverslips. To minimize cross-talk between the different fluorescence labels, the multi-tracking setting of the Zeiss Zen software was used, which sequentially illuminated and detected one fluorophore at a time [194]. The image settings were saved and re-used for subsequent experiments in order to compare differences in virus and antibody intensity levels.

Images were processed using MetaMorph (Molecular Devices) program. Maximum projection images were used to include fluorescence signals from all the Z planes. Viral particles on the surface of target cells were identified using SNAP-ICAM-1 signals. Orientation on the outer cell membrane surface was determined by Phalloidin staining signals (see S3 Fig.). Regions of interests (ROIs) were traced around ICAM-1 SNAP Surface Alexa 546 signals. The ROIs perimeters were configured to define where fluorescent signals fell to apparent background. Such boundaries were more expansive than the full width at half maximum measures of point spread function (200–250 nm for our instrument), but were desirable as a means to include all the intensity signals for unbiased and comprehensive comparisons. This approach was rehearsed using 0.1μm carboxylate-modified red microspheres (FluoSpheres; Invitrogen), which have similar excitation and emission spectra as the SNAP-ICAM-1 label (Ex_max_: 540nm, Em_max_: 560nm) and a diameter matching the lower end of the size range [138, 139] reported for HIV particles (0.145–0.181 μm). As such, the microspheres provided a serviceable gauge, although they are designed to be intensely fluorescent with little or no photobleaching and are likely to exhibit greater image resolution compared to the randomly incorporated SNAP-ICAM-1 label on virus surfaces. Configured as stated above, ROIs determined for microspheres settled on to target cells exhibited a median diameter of 0.72 ± 0.09 μm. The ROIs for putative cell-bound virions exhibited a similar median diameter of 1.32 ± 0.17 μm; ROIs for virions bound to coverslips exhibited a median diameter of 1.25 ± 0.15 um. These concordant sizes indicated that in general single entities versus large “clumps” of virions were interrogated on target cell surfaces. However, the possibility that we occasionally surveyed multiple particles located in close proximity cannot be completely eliminated.

To correct for background fluorescence, each ROI meeting the above criteria was compared to a mock ROI. These were randomly selected regions of equivalent diameter on condition-matched cells that did not have apparent SNAP-ICAM-1 signals. Background intensity levels in all 3 color channels were recorded and subtracted from corresponding ROI measures. For the displayed images, each fluorescent channel was deconvolved using the nearest neighbor algorithm in MetaMorph to decrease the noise in the system.

SNAP-ICAM-1, CLIP-Vpr, and antibody relative intensity values were exported from MetaMorph as excel files and graphed using GraphPad Prism (version 5.04 for Windows) or SigmaPlot (version 12.0 for Windows). For all data points to appear in the log scale, intensity values less than 1 were amended to be equal to 1. Since the virus and antibody intensity signals did not have a normal distribution, the non-parametric Kruskal-Wallis was performed for statistical analysis, using GraphPad Prism.

To quantify the cell-bound particle and antibody signals, fluorescence intensity data of HIV-1_JRFL_ (SNAP-ICAM-1 & CLIP-Vpr), and test Mabs were imported into the flow cytometry software, FlowJo X. This allowed standard Boolean gating of the data in order to examine differences in the various treatment groups. To achieve this, a same day experimental set was normalized, and then log transformed to offset the treatment of the input data as log values. Finally, the numbers were multiplied by 1000 to spread the data set to the capacity of the software [the number of pseudocolor palettes of a 12-bit image (2^12^ = 4096)].

### Direct STochastic Optical Reconstruction Microscopy (dSTORM)

Conditions similar to the confocal experiments were used for direct stochastic optical reconstruction microscopy (dSTORM) experiments. 4.0 x 10^5^ TZM-bl cells were seeded on 20-mm^2^ glass-bottom MatTek dishes (MatTek Corp., Ashland, MA) and incubated overnight at 37°C in 5% CO_2_. The next day, HIV-1_JRFL_ pseudoviruses expressing SNAP-ICAM-1 and CLIP-Vpr were fluorescently labeled with SNAP-Surface Alexa 546 and CLIP-Cell Alexa Fluor 360, respectively as described above. The TZM-bl cells were co-cultured with 0.5mL of 3 x 10^6^ TCID50/mL of these fluorescently-labeled HIV-1_JRFL_, and with 5μg/mL Alexa 647-conjugated OKT4 (a non HIV neutralizing anti-CD4 antibody) for 30 minutes at 37°C, then immediately fixed with 4% Paraformaldehyde for 15 minutes at room temperature. Non-specific interactions were blocked by incubating coverslips with 10% normal goat serum and 100μg/mL non-specific IgG1 solution for 30 minutes at room temperature. Epitopes of interest were probed with 5μg/mL Alexa Fluor 488-conjugated antibodies for 30 minutes at room temperature. Dishes were then post-fixed in 4% Paraformaldehyde for 15 minutes at room temperature and stored in 1X PBS at 4°C until imaging.

### dSTORM Image Acquisition and Analysis

Three-color, 3D dSTORM imaging was carried out using the Nikon N-STORM microscope (Nikon Instruments Inc., Melville, NY). Using the 100X CFI Apo TIRF oil-immersion objective (1.49 NA), 256 by 256 XY scans, 0.165 μm/pixel size, were acquired, and 3D images were obtained using the astigmatism method of 3D localization [113]. The 647nm, 561nm, and 488nm laser lines were used to excite Alexa 647-conjugated OKT4, SNAP-ICAM-1 labeled with SNAP Surface Alexa 546, and Alexa 488-conjugated Mabs, respectively. The 405 laser was used to obtain TIRF images of the CLIP-Vpr Alexa 360 signal. The oxygen-scavenging imaging buffer was 14mg glucose oxidase and 50μL of 17mg/mL catalase (Sigma) in 200μL Component A (10mM Tris, 50mM NaCl); Component B (50mM Tris-HCl, 10mM NaCl, 10% glucose); and 1M cysteamine (MEA).

Single molecule fitting and Gaussian images were rendered using the N-STORM software NIS Elements (version 4.30.01). The localization precision for all three fluorophores was determined to be 20 nm using full width at half maximum (FWHM), with a 50 nm axial resolution. After high resolution images were obtained, ROIs were defined around single virions with ICAM-1 and Mab signals within 200nm. Virus-bound Mab signals and the number of localized signals were recorded.

### Antibody Quantitation Based on Localized Events

The calibration method was designed to equate localized events (Alexa Fluor 488 fluorophore signal “blinks”) with a defined number of dye-conjugated antibody molecules within a superresolution ROI. To do this, MatTek dishes were coated with 5μg/mL D7324 in 1X PBS overnight at room temperature and were used to capture 0.5μg/mL full length single chain (FLSC), a monomeric gp120-sCD4 complex stably expressing CD4i epitopes [174]. Thus, the substrate-bound bivalent antibody in an ROI could theoretically capture one or two FLSC antigens; which in turn could capture one or two conjugated Mabs. Importantly, the capture format was selected because it preserves the native structure of the target antigen by avoiding chemical modifications or direct adsorbance to substrate, either of which can perturb epitope presentation. Alexa Fluor 488-labeled Mabs 2G12, A32, or 17b (5μg/mL) were incubated with the captured FLSC for 30 minutes at room temperature, fixed and imaged by dSTORM. ROIs were circumscribed around distinct Gaussian signals, and the corresponding number of localized events was extracted. The number of localized events measured within an ROI was then taken to reflect the presence of one or two conjugated Mabs. As this qualification applied to all target epitopes, cross-comparisons of Mabs tested under identical conditions were feasible.

## Supporting Information

S1 Table Statistical comparison of Vpr (low) populations. The Vpr (low) populations were defined as the bottom 5th percentile readings of HIV_JRFL_—TZM-bl co-cultures at 5 minutes. The 5-minute-threshold also defined the Vpr (low) populations in the remaining time points. A two-sample Kolmogorov-Smirnov test was used to compare Vpr (low) populations between adjacent time points. P-values were adjusted using a Bonferroni multiple-test correction (factor = 4). The Vpr (low) populations show significant differences between all adjacent time points tested, except for the 15 vs 30 minute-time point comparison.
(TIFF)

S1 Fig Representative images of HIV_JRFL_ virions attached to poly-l-lysine—coated coverglass. HIV_JRFL_ virions were treated with membrane-impermeable SNAP-Surface Alexa Fluor 546 (Red) and membrane-permeable CLIP-Cell Alexa Fluor 360 (Blue) to fluorescently tag SNAP-ICAM-1 and CLIP-Vpr, respectively. Labeled virions were adhered to poly-l-lysine-coated coverglass for 2 hours at 4°C. Gp120 epitope exposure was probed with Alexa 488 (green)-conjugated Mabs b12, 2G12, A32, C11 or 17b. Synagis was used as a negative control. The dashed yellow lines around virus particles represent example ROIs selected as described in **Methods**. Scale bar = 1μm.
(TIFF)

S2 Fig Bound HIV_JRFL_ virions measured as regions of interest do not present free viral antigens. (A) HIV_JRFL_ virions tagged with SNAP-ICAM-1 (red) and CLIP-Vpr (blue) were attached to poly-l-lysine coated coverglass for 2 hours at 4°C. Virion degradation was assessed by Alexa 488 (green)-conjugated monoclonal anti-p24 (Abcam Ab9071) antibody; the presence of monomeric gp120 was probed with polyclonal D7324 antibodies against the gp120 C terminus. Tests with anti-p24 antibody were made before (pre) and after (post) viral membrane permeabilization with 0.2% Triton X-100. The latter serves as a positive control for the presence of HIV_JRFL_ capsid in the intra-viral space. Scale bar = 1μm. (B) Tagged virions were attached to TZM-bl cells for 120 minutes, fixed and probed with above antibodies prior to permeabilization with 0.2% Triton X-100 and staining of peripheral actin with Phalloidin for the identification of virions on the cell surface as described in **Methods**. HIV_JRFL_ with [Vpr(+)] or without [(Vpr(0)] Vpr signals were selected to assess gp120 dissociation and capsid protein exposure in these subpopulations. The dashed yellow lines depict representative ROIs selected as described in **Methods**. Scale bar = 1μm.
(TIFF)

S3 Fig Cortical actin staining to distinguish cell surface bound HIV_JRFL_. Target cells were used to capture HIV_JRFL_ virions, which were then treated with test Mabs and fixed. After Mab staining and fixing, the cells were then permeabilized to label cortical actin with Alexa-647-conjugated phalloidin (see Methods). Extracellular ROIs were selected based on the phalloidin staining pattern. (A) Representative image of HIV_JRFL_ bound to TZM-bl cells. Phalloidin staining is shown in cyan, virus-associated SNAP-ICAM-1 in red. Corresponding axial (Z) images are shown in the lower panels, with the arrow pointing toward the top of the cell. Scale bar = 5μm. (B) Close up image of yellow box in (A) indicating how the cell periphery is defined based on phalloidin signals viewed in lateral and axial orientations (dashed white line); and an ROI is selected based on calibrated size SNAP-ICAM-1 signal (dashed yellow line). Corresponding axial (Z) images are shown in the lower panels, with the arrow pointing to the upper surface of the cell. Scale bar = 1μm.
(TIFF)

S4 Fig Representative fluorescence signals from HIV_JRFL_ virions settled on to HIV receptor-negative HeLa cells. SNAP-Alexa 546 (Red) and CLIP-Alexa 360 (blue) tagged HIV_JRFL_ virions were settled on to HeLa cells for 30 minutes. Gp120 epitope exposure was probed with Alexa 488 (green)-conjugated neutralizing Mab 2G12, or CD4i Mabs A32, C11, or 17b, as well as the negative control Synagis. Scale bar = 1μm. ROIs (yellow) were selected using the methods employed with TZM-bl cells.
(TIFF)

S5 Fig Gp120 epitope exposure on TZM-bl cell-bound HIV_JRFL_ virions under membrane fusion-permissive or non-permissive conditions. Relative antibody intensity signals from at least 200 ROIs/condition were collected for particles bound to TZM-bl cells for the indicated periods of time at either 37°C (black) or 4°C (blue), which facilitate or prohibit membrane fusion, respectively. Red lines represent the geometric mean of the data; green bars indicate standard errors.
(TIFF)

S6 Fig Three dimensional superresolution images of TZM-bl—bound HIV_JRFL_ probed with Mab A32. Alexa 488 (green)-conjugated Mabs A32 (A) and 17b (B) were tested in the same manner as Mab 2G12 in Fig. 7. TZM-bl surface CD4 is stained with Alexa 647-tagged OKT4 (red); the virion surface is marked by ICAM-1 tagged with SNAP-Alexa546 (Blue). Top left: XY images. Scale bar = 0.1μm; top right: Axial views of the dSTORM image with the color channels separated as well as merged together. The Z line is pointing upwards away from the cell. The bottom images match the ones above, but with color coded Z position scaling as in Fig. 7.
(TIFF)

S7 Fig Standardization of antibody fluorescence signals. A gp120-CD4 fusion protein (FLSC), was captured on D7324-coated cover glass (see Methods) and reacted with Alexa 488-conjugated Mabs 2G12 (red), A32 (green), and 17b (blue) were allowed to bind for 30 minutes at room temperature. (A) The number of localized events generated from superresolution ROIs (Mab 2G12, N = 107; Mab A32, N = 113; or Mab 17b, N = 103) are shown. Black bars indicate the geometric mean and standard errors. The two-tailed Mann-Whitney test was used to perform pairwise comparisons of localized events measured with each Mab. (B) Histogram of the number of localized events from ROIs containing test Mabs 2G12 (red), A32 (green), and 17b (blue), tested as shown in (A).
(TIFF)

S1 Movie Rotation of the 3D projection image shown in Fig. 7. HIV_JRFL_ virion attached to a TZM-bl cell, with cell surface CD4 stained with Alexa 647-conjugated, anti-CD4 antibody OKT4 (red), virion surface marked by ICAM-1 tagged with membrane-impermeable SNAP-Alexa 546 (Blue), and gp120 stained by Alexa 488-conjugated Mab 2G12 (green).
(AVI)

S2 Movie Rotation of the 3D projection image shown in S6A Fig. HIV_JRFL_ virion attached to a TZM-bl cell, with cell surface CD4 stained with Alexa 647-conjugated, anti-CD4 antibody OKT4 (red), virion surface marked by ICAM-1 tagged with membrane-impermeable SNAP-Alexa 546 (Blue), and gp120 stained by Alexa 488-conjugated Mab A32 (green).
(AVI)

S3 Movie Rotation of the 3D projection image shown in S6B Fig. HIV_JRFL_ virion attached to a TZM-bl cell, with cell surface CD4 stained with Alexa 647-conjugated, anti-CD4 antibody OKT4 (red), virion surface marked by ICAM-1 tagged with membrane-impermeable SNAP-Alexa 546 (Blue), and gp120 stained by Alexa 488-conjugated Mab 17b (green).
(AVI)
