# Correction: Antigenic Properties of the Human Immunodeficiency Virus Envelope Glycoprotein Gp120 on Virions Bound to Target Cells

**DOI:** 10.1371/journal.ppat.1004990

**Published:** 2015-06-15

**Authors:** The PLOS Pathogens Staff

## Notice of Republication

This article was republished on May 22, 2015, to correct Figures 5 and 6, which were both resized during the typesetting process. The publisher apologizes for the errors. Please download this article again to view the correct version.
