# Supplementary figures and images for: Antigenic Properties of the Human Immunodeficiency Virus Envelope Glycoprotein Gp120 on Virions Bound to Target Cells
Source: PLoS Pathog. 2015 Mar 25;11(3):e1004772. doi: 10.1371/journal.ppat.1004772 (PMC4373872; doi:10.1371/journal.ppat.1004772)

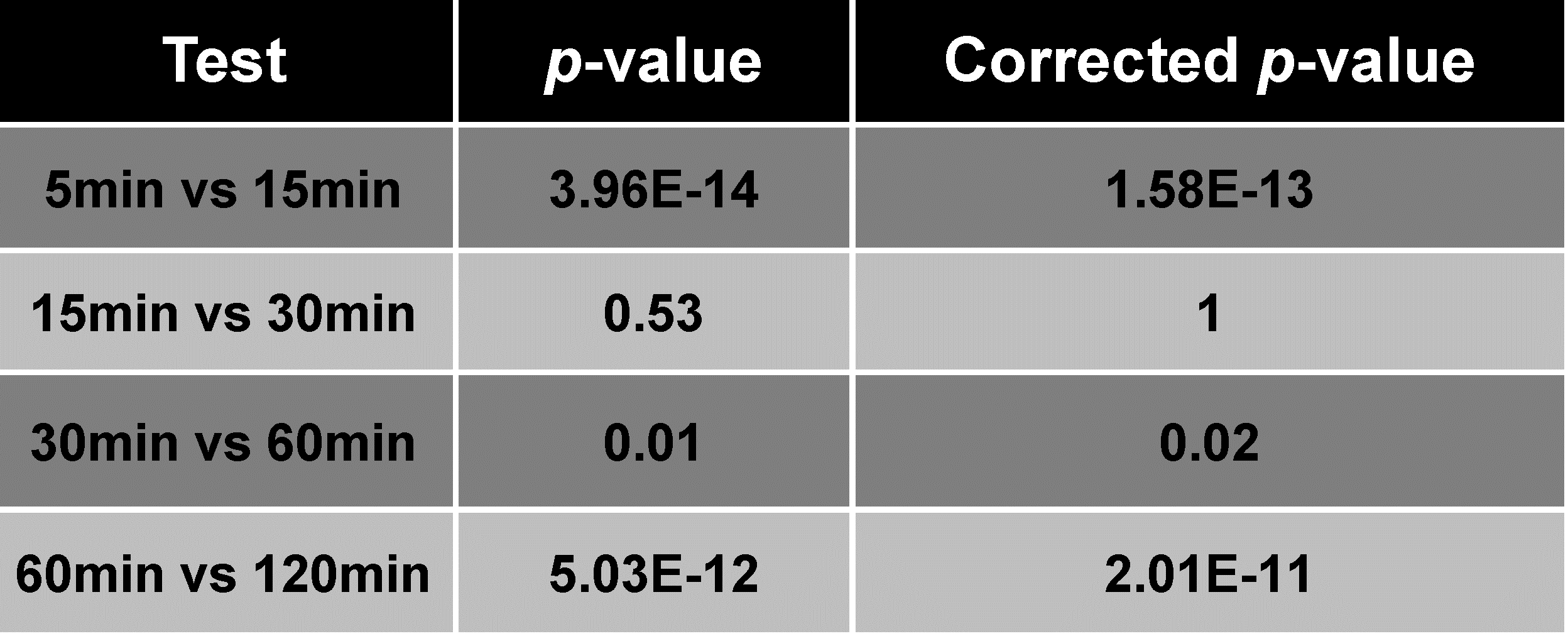

Supplement: S1 Table — The Vpr (low) populations were defined as the bottom 5th percentile readings of HIVJRFL—TZM-bl co-cultures at 5 minutes. The 5-minute-threshold also defined the Vpr (low) populations in the remaining time points. A two-sample Kolmogorov-Smirnov test was used to compare Vpr (low) populations between adjacent time points. P-values were adjusted using a Bonferroni multiple-test correction (factor = 4). The Vpr (low) populations show significant differences between all adjacent time points tested, except for the 15 vs 30 minute-time point comparison. (TIFF) [file ppat.1004772.s001.tiff]

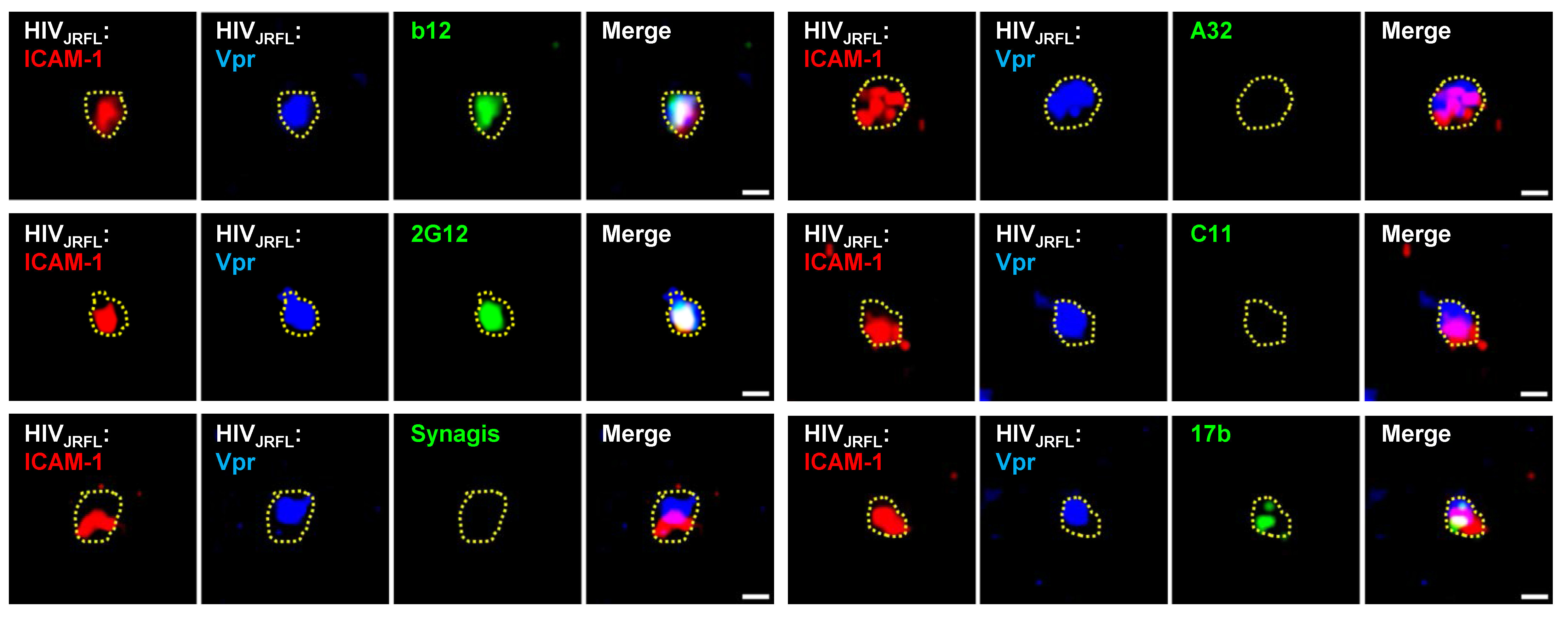

Supplement: S1 Fig — HIVJRFL virions were treated with membrane-impermeable SNAP-Surface Alexa Fluor 546 (Red) and membrane-permeable CLIP-Cell Alexa Fluor 360 (Blue) to fluorescently tag SNAP-ICAM-1 and CLIP-Vpr, respectively. Labeled virions were adhered to poly-l-lysine-coated coverglass for 2 hours at 4°C. Gp120 epitope exposure was probed with Alexa 488 (green)-conjugated Mabs b12, 2G12, A32, C11 or 17b. Synagis was used as a negative control. The dashed yellow lines around virus particles represent example ROIs selected as described in Methods. Scale bar = 1μm. (TIFF) [file ppat.1004772.s002.tiff]

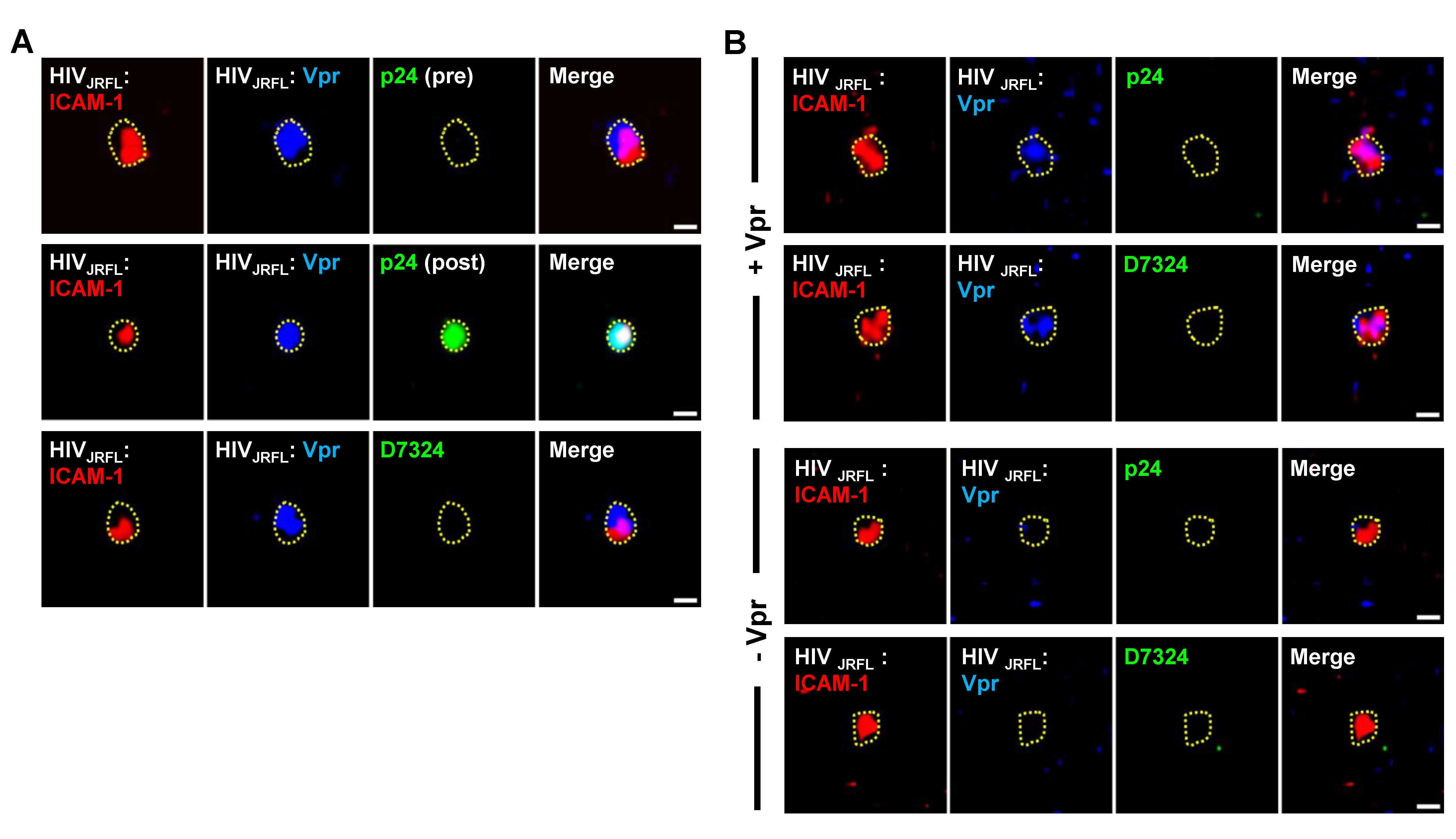

Supplement: S2 Fig — (A) HIVJRFL virions tagged with SNAP-ICAM-1 (red) and CLIP-Vpr (blue) were attached to poly-l-lysine coated coverglass for 2 hours at 4°C. Virion degradation was assessed by Alexa 488 (green)-conjugated monoclonal anti-p24 (Abcam Ab9071) antibody; the presence of monomeric gp120 was probed with polyclonal D7324 antibodies against the gp120 C terminus. Tests with anti-p24 antibody were made before (pre) and after (post) viral membrane permeabilization with 0.2% Triton X-100. The latter serves as a positive control for the presence of HIVJRFL capsid in the intra-viral space. Scale bar = 1μm. (B) Tagged virions were attached to TZM-bl cells for 120 minutes, fixed and probed with above antibodies prior to permeabilization with 0.2% Triton X-100 and staining of peripheral actin with Phalloidin for the identification of virions on the cell surface as described in Methods. HIVJRFL with [Vpr(+)] or without [(Vpr(0)] Vpr signals were selected to assess gp120 dissociation and capsid protein exposure in these subpopulations. The dashed yellow lines depict representative ROIs selected as described in Methods. Scale bar = 1μm. (TIFF) [file ppat.1004772.s003.tiff]

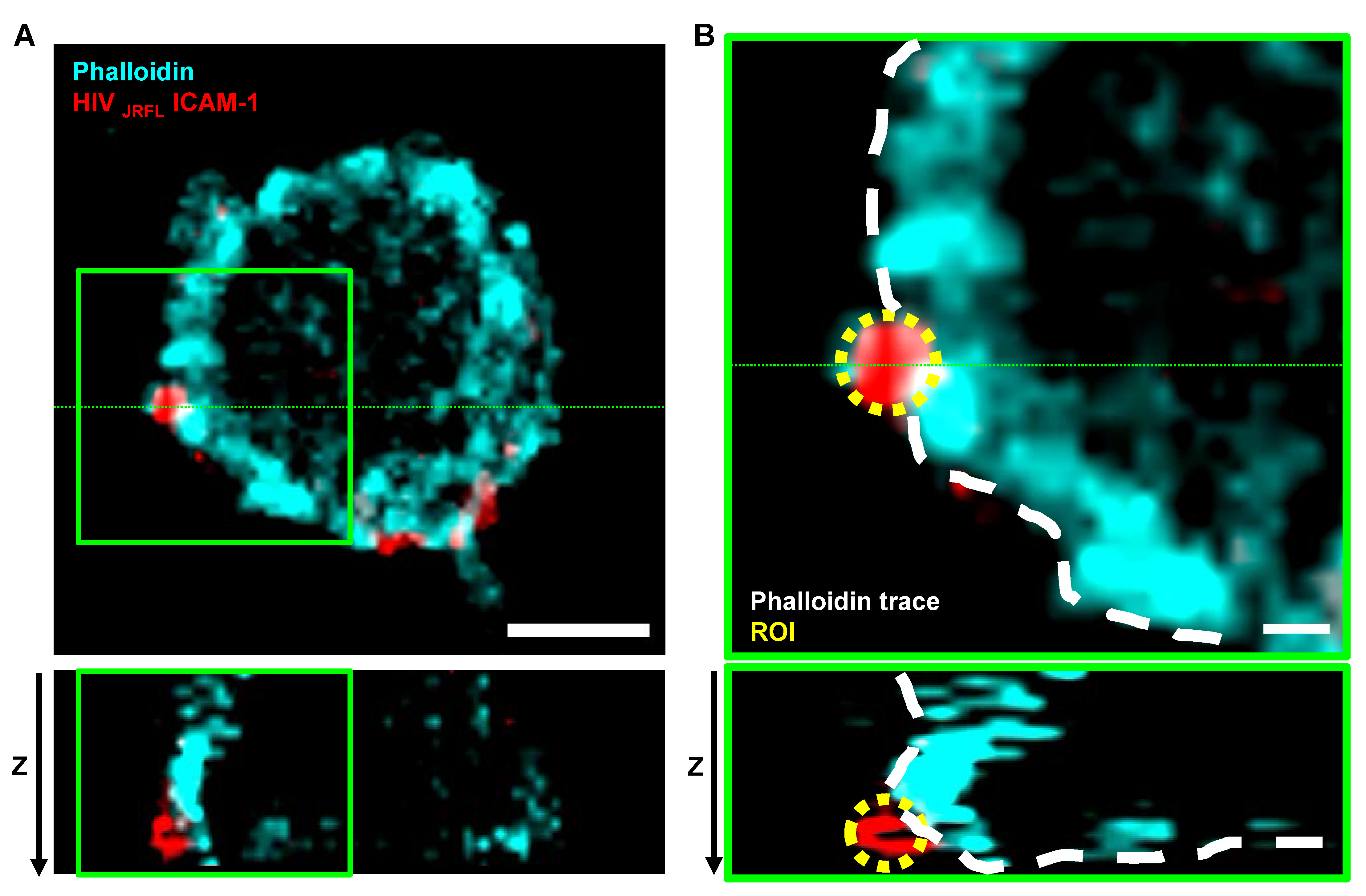

Supplement: S3 Fig — Target cells were used to capture HIVJRFL virions, which were then treated with test Mabs and fixed. After Mab staining and fixing, the cells were then permeabilized to label cortical actin with Alexa-647-conjugated phalloidin (see Methods). Extracellular ROIs were selected based on the phalloidin staining pattern. (A) Representative image of HIVJRFL bound to TZM-bl cells. Phalloidin staining is shown in cyan, virus-associated SNAP-ICAM-1 in red. Corresponding axial (Z) images are shown in the lower panels, with the arrow pointing toward the top of the cell. Scale bar = 5μm. (B) Close up image of yellow box in (A) indicating how the cell periphery is defined based on phalloidin signals viewed in lateral and axial orientations (dashed white line); and an ROI is selected based on calibrated size SNAP-ICAM-1 signal (dashed yellow line). Corresponding axial (Z) images are shown in the lower panels, with the arrow pointing to the upper surface of the cell. Scale bar = 1μm. (TIFF) [file ppat.1004772.s004.tiff]

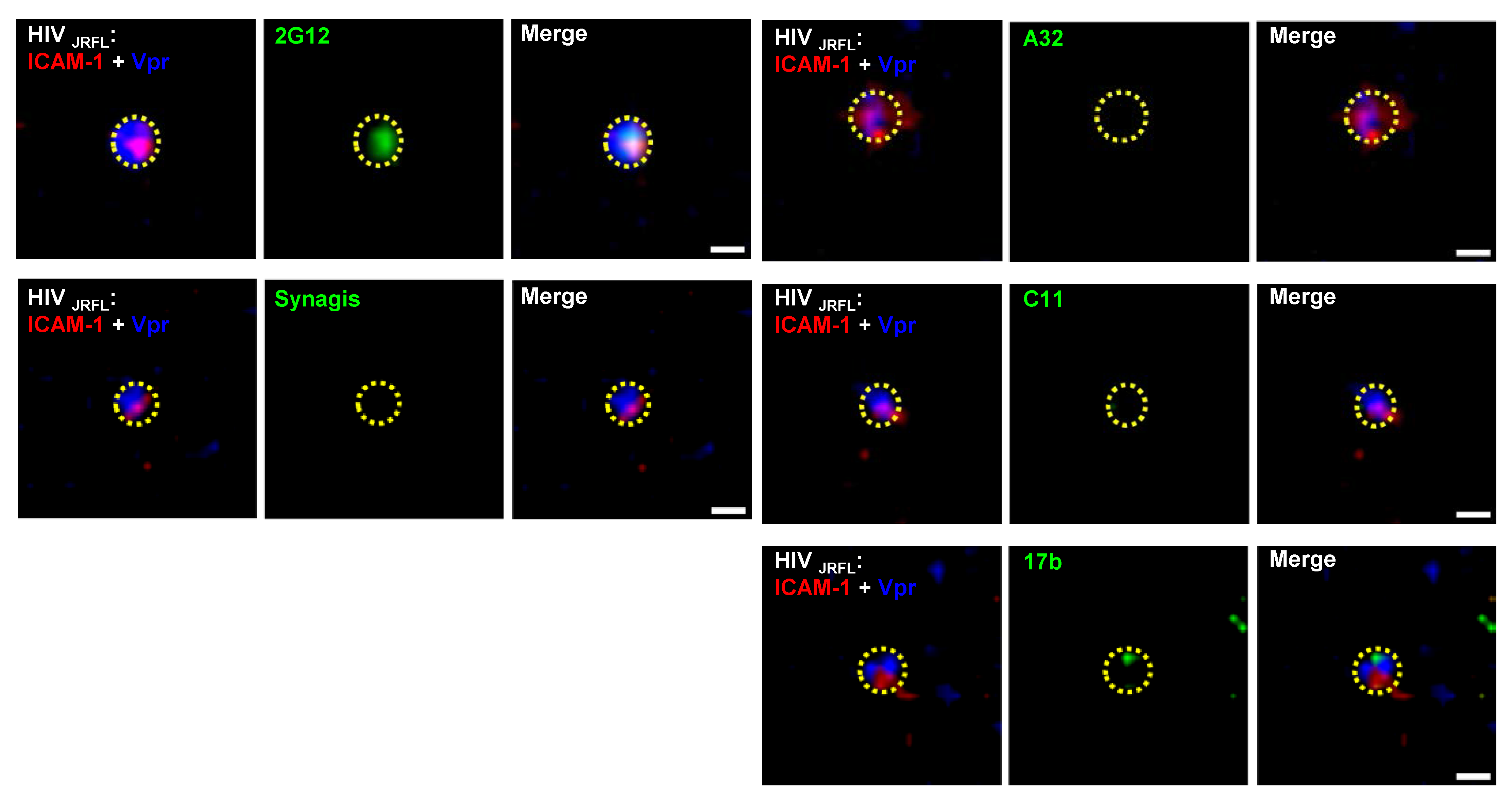

Supplement: S4 Fig — SNAP-Alexa 546 (Red) and CLIP-Alexa 360 (blue) tagged HIVJRFL virions were settled on to HeLa cells for 30 minutes. Gp120 epitope exposure was probed with Alexa 488 (green)-conjugated neutralizing Mab 2G12, or CD4i Mabs A32, C11, or 17b, as well as the negative control Synagis. Scale bar = 1μm. ROIs (yellow) were selected using the methods employed with TZM-bl cells. (TIFF) [file ppat.1004772.s005.tiff]

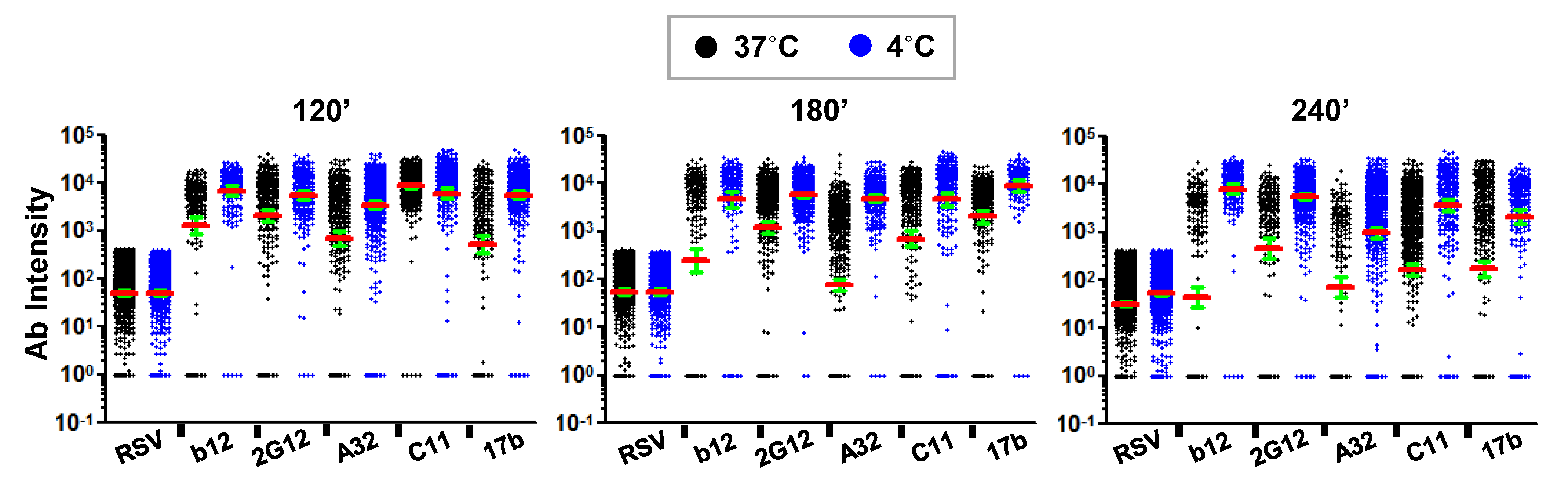

Supplement: S5 Fig — Relative antibody intensity signals from at least 200 ROIs/condition were collected for particles bound to TZM-bl cells for the indicated periods of time at either 37°C (black) or 4°C (blue), which facilitate or prohibit membrane fusion, respectively. Red lines represent the geometric mean of the data; green bars indicate standard errors. (TIFF) [file ppat.1004772.s006.tiff]

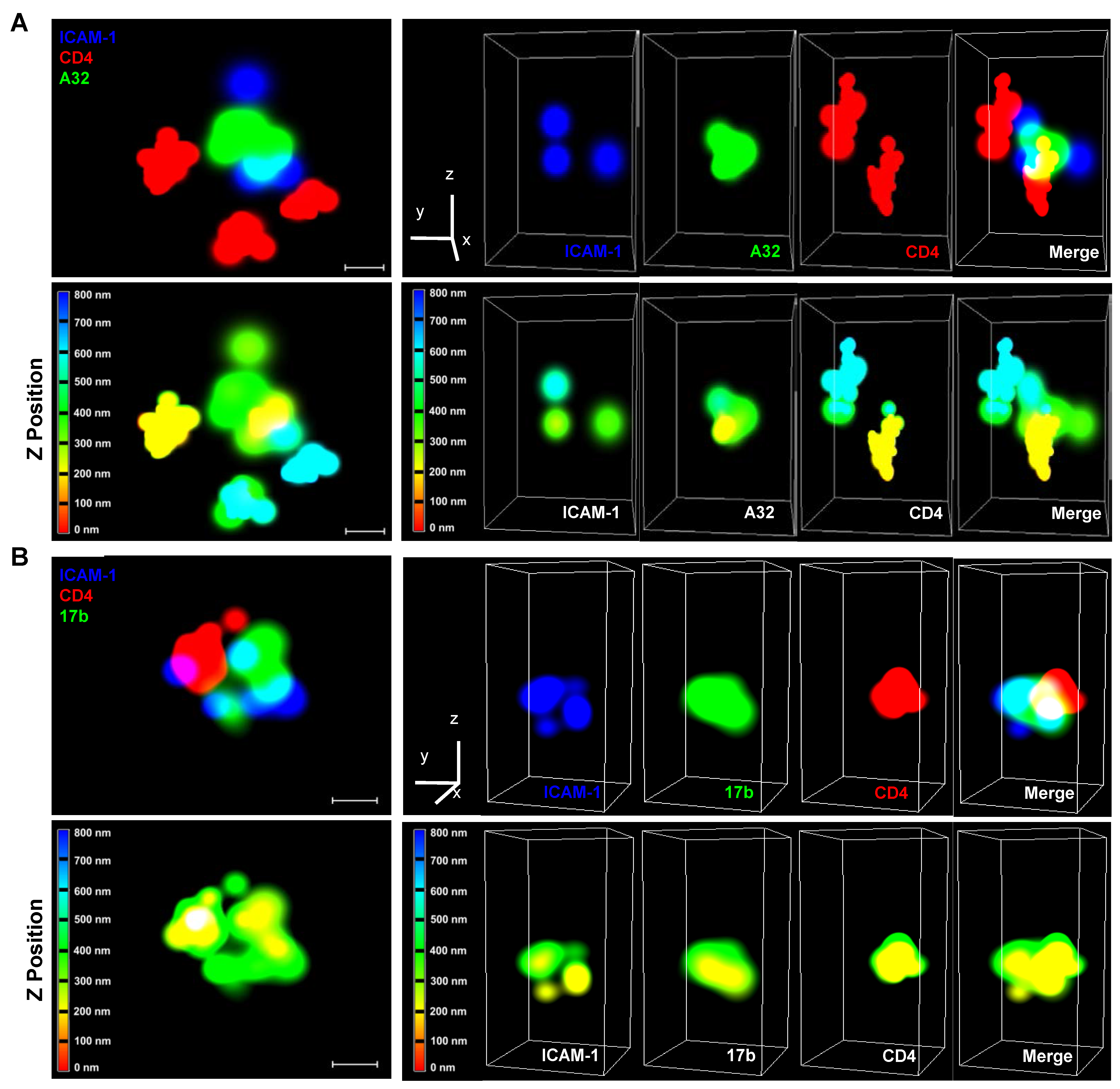

Supplement: S6 Fig — Alexa 488 (green)-conjugated Mabs A32 (A) and 17b (B) were tested in the same manner as Mab 2G12 in Fig. 7. TZM-bl surface CD4 is stained with Alexa 647-tagged OKT4 (red); the virion surface is marked by ICAM-1 tagged with SNAP-Alexa546 (Blue). Top left: XY images. Scale bar = 0.1μm; top right: Axial views of the dSTORM image with the color channels separated as well as merged together. The Z line is pointing upwards away from the cell. The bottom images match the ones above, but with color coded Z position scaling as in Fig. 7. (TIFF) [file ppat.1004772.s007.tiff]

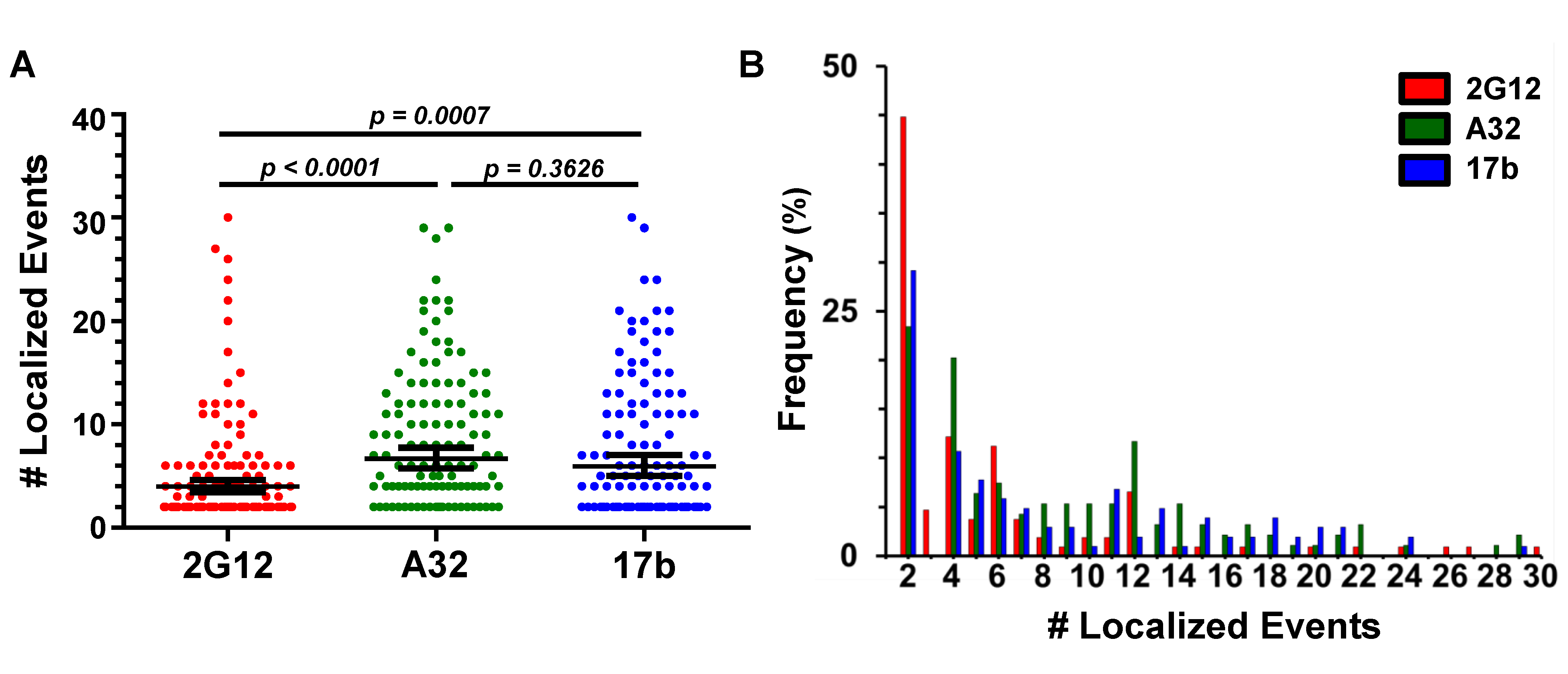

Supplement: S7 Fig — A gp120-CD4 fusion protein (FLSC), was captured on D7324-coated cover glass (see Methods) and reacted with Alexa 488-conjugated Mabs 2G12 (red), A32 (green), and 17b (blue) were allowed to bind for 30 minutes at room temperature. (A) The number of localized events generated from superresolution ROIs (Mab 2G12, N = 107; Mab A32, N = 113; or Mab 17b, N = 103) are shown. Black bars indicate the geometric mean and standard errors. The two-tailed Mann-Whitney test was used to perform pairwise comparisons of localized events measured with each Mab. (B) Histogram of the number of localized events from ROIs containing test Mabs 2G12 (red), A32 (green), and 17b (blue), tested as shown in (A). (TIFF) [file ppat.1004772.s008.tiff]
